# Rac1 palmitoylation is required for cardiac stress adaptation and regulation of protein kinase A signaling

**DOI:** 10.1172/jci.insight.193733

**Published:** 2025-09-09

**Authors:** James P. Teuber, Rachel E. Scissors, Arasakumar Subramani, Nageswara Madamanchi, Matthew J. Brody

**Affiliations:** 1 Department of Pharmacology, and; 2 Division of Cardiovascular Medicine, Department of Internal Medicine, University of Michigan, Ann Arbor, Michigan, USA.

**Keywords:** Cardiology, Cell biology, Cell stress, Mouse models, Signal transduction

## Abstract

Cardiac hypertrophy is a common adaptation to cardiovascular stress and often a prelude to heart failure. We examined how *S*-palmitoylation of the small GTPase, Ras-related C3 botulinum toxin substrate 1 (Rac1), impacts cardiomyocyte stress signaling. Mutation of the Cys-178 palmitoylation site impaired activation of Rac1 when overexpressed in cardiomyocytes. Cardiomyocyte-specific Rac1 conditional knockin (Rac1^cKI^) mice expressing a Rac1^C178S^ mutant protein exhibited normal cardiac structure and function but developed more severe cardiac hypertrophy in response to angiotensin II (AngII) infusion, cardiomyocyte-specific overexpression of AngII type 1 receptor (AT1R), and cardiac pressure overload. Moreover, pressure overload and AT1R overexpression evoked cardiac failure phenotypes in Rac1^cKI^ mice not observed in controls. Mechanistically, Rac1^cKI^ hearts and cardiomyocytes genetically resistant to Rac1 *S*-palmitoylation had a profound increase in protein kinase A (PKA) substrate phosphorylation in response to acute β-adrenergic stimulation, as did Rac1^cKI^ hearts subjected to chronic AngII treatment, AT1R overexpression, or pressure overload that correlates with more advanced heart failure phenotypes. This was not associated with increased PKA enzymatic activity, suggesting potential deficits in phosphatase activity at PKA-regulated phospho-sites. Taken together, this study suggests Rac1 *S*-palmitoylation dampens adrenergic drive and PKA-dependent modulation of the phospho-proteome in response to cardiovascular stress, revealing essential functions for *S*-acylated Rac1 in cardiac adaptation.

## Abstract 2

Modification of Rac1 by protein palmitoylation restrains hyeractive protein kinase A (PKA) activity and pathological cardiac hypertrophy and adverse remodeling in response to stress.

## Graphical abstract

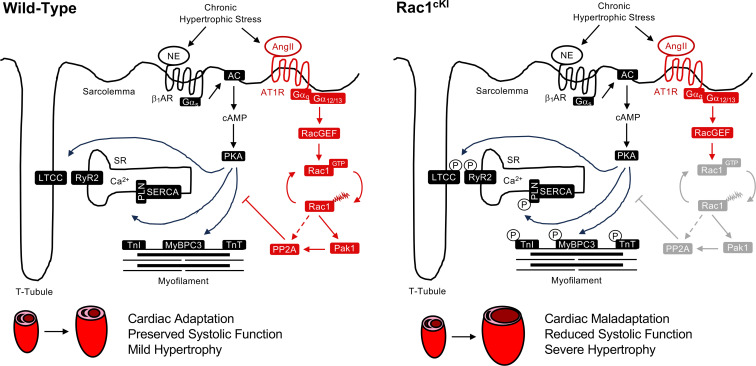

## Introduction

Heart disease is the leading cause of death globally and poses an enormous public health and economic burden (1–3). Heart failure alone afflicts more than 6 million Americans, costing more than $30 billion and leading to roughly 800,000 hospitalizations annually (2, 4, 5). Cardiac hypertrophy, the enlargement of the heart in response to an increased workload, is an initially adaptive response to preserve cardiac output (6–8). However, in the presence of chronic stressors, such as enhanced sympathetic activity and renin-angiotensin system (RAS) activation, cardiac hypertrophy often progresses to maladaptive cardiac remodeling, ventricular dilation, declined cardiac function, and ultimately heart failure (6, 8, 9). Activation of RAS and elevated levels of circulating angiotensin II (AngII) primarily act on the vasculature to promote vasoconstriction but also directly on the heart to promote hypertrophy and fibrosis through activation of AngII type 1 receptors (AT1Rs) on cardiomyocytes and fibroblasts, respectively. As a result, these pathways have long been targets of heart failure therapeutics (10–13).

Elevated levels of circulating catecholamines and enhanced sympathetic activity promote inotropy through enhanced protein kinase A–mediated (PKA-mediated) phosphorylation of myofilament proteins and calcium cycling machinery that aids in maintenance of cardiac output. However, chronic elevation of β-adrenergic signaling in cardiomyocytes becomes maladaptive and accelerates the progression to heart failure, necessitating widespread clinical use of beta blockers in heart failure patients (13–19). Moreover, PKA activity is required for pathologic cardiac hypertrophy and failure in response to left ventricular pressure overload (20), suggesting an essential role for PKA in the pathogenesis of adverse cardiac remodeling and heart failure progression. Indeed, the cardiomyocyte has intrinsic mechanisms to dampen the inotropic response to sustained β-adrenergic signaling, including protein phosphatase activity that antagonizes phosphorylation of substrates by PKA (18, 21, 22). Thus, RAS activation and enhanced sympathetic drive, although initially adaptive to preserve cardiac performance and tissue perfusion, precipitate a vicious cycle of cardiac maladaptation that progresses to heart failure. The integration and regulation of intracellular signaling pathways downstream of chronically stimulated β adrenergic receptors (βARs) and AT1Rs in cardiomyocytes, and how this contributes to the progression of heart failure despite routine pharmacological targeting of both pathways (13, 15), remains incompletely understood.

Ras-related C3 botulinum toxin substrate 1 (Rac1) is a small GTPase of the Rho family that functions as a molecular switch to mediate cell adhesion, migration, and proliferation, oxidative stress, and remodeling of the actin cytoskeleton (23–26). In cardiomyocytes, Rac1 signaling is required for proper cardiac development (27–29) and hypertrophy of the adult heart in response to increased afterload (26, 30). Overexpression of a constitutively active Rac1 mutant results in dilated cardiomyopathy (31, 32). Conversely, cardiomyocyte-specific deletion or knockdown of Rac1 protects against cardiac hypertrophy in response to AngII infusion (26) or left ventricular pressure overload induced by transverse aortic constriction (TAC) (30), respectively. These findings suggest Rac1 signaling in cardiomyocytes promotes pathologic cardiac growth and decompensation. A proteomic screen recently identified Rac1 as a substrate of the Golgi *S*-acyltransferase, zinc finger Asp-His-His-Cys domain–containing 3 (zDHHC3) (33). Transgenic overexpression of zDHHC3 in cardiomyocytes results in severe heart failure, which is associated with increased Rac1 *S*-palmitoylation and GTP loading (33). This suggests that modification of Rac1 by *S*-palmitoylation may promote compartmentalized Rac1 activity in cardiomyocytes to elicit maladaptive hypertrophy (26, 32).

*S*-palmitoylation, also known as *S*-acylation, is a reversible posttranslational lipid modification of cysteine residues on substrate proteins with a saturated fatty acid, most commonly 16-carbon palmitate (34, 35), providing a dynamic mechanism to regulate association of cytosolic proteins with membrane-associated receptors, effectors, or signalosomes (34–36). *S*-palmitoylation is catalyzed by the zDHHC family of *S*-acyltransferases and can be reversed by specific cytosolic serine hydrolases that have *S*-acyl protein thioesterase (APT) activity, such as of the lysophospholipase family (APT-1 and APT-2 encoded by *Lypla1* and *Lypla2* genes) and/β-hydroxylase domain–containing (ABHD-containing) 17 subfamily (ABHD17A, -B, and -C) of enzymes (8, 34, 35). However, the functions of these enzymes in the heart and their role in cardiac stress adaptation remain ill defined.

In addition to the permanent isoprenoid lipid modifications on the C-terminus of all small GTPases essential for their processing and trafficking, some small GTPases such as H-Ras and N-Ras are also additionally modified by *S*-palmitoylation. This modification provides an enzymatically regulated lipid-based tag that facilitates deployment of substrates to membrane microdomains and is ideally suited for superimposition of molecular signal transduction circuitry, such as a GTPase cycle, on the spatial landscape of intracellular membranes (25, 36–38). Rac1 *S*-palmitoylation at Cys-178 within the C-terminal hypervariable region is required for partitioning into liquid-ordered membranes and actin cytoskeleton remodeling in COS-7 cells (25). It is not known, however, how *S*-palmitoylation impacts compartmentalized Rac1 signaling activity and its downstream effectors in cardiomyocytes, which have a relatively static cytoskeleton, limited migration and proliferation, and unique structurally and functionally distinct plasma membrane domains.

To examine the role of *S*-palmitoylation of Rac1 in cardiomyocyte stress-responsive signaling and cardiac hypertrophy in vivo, we generated conditional knockin mice with cardiomyocyte-specific mutation of the Cys-178 palmitoylation site (Rac1^cKI^) and subjected them to several models of chronic pathological hypertrophic stress to evaluate their propensity for the development of heart failure. Surprisingly, Rac1^cKI^ mice were phenotypically normal in the absence of stress but exhibited more severe cardiac hypertrophy and functional decompensation when subjected to multiple models of cardiac hypertrophy. Rac1^cKI^ hearts exhibit hyperphosphorylation of PKA substrates that is associated with more advanced heart failure phenotypes in response to AngII infusion, transgenic AT1R overexpression, or TAC-induced pressure overload and in response to acute β-adrenergic stimulation with isoproterenol. These findings indicate a role for palmitoylated Rac1 in dampening cardiomyocyte PKA signaling. Previous evidence has highlighted roles for the Rac1 effector protein, p21-activated kinase 1 (Pak1), in the regulation of protein phosphatase 2A (PP2A) activity in cardiomyocytes, which has critical functions in modulating contractility and calcium cycling primarily through dephosphorylation of PKA substrates (22, 39, 40). RNA sequencing (RNA-seq) revealed unique downregulation of the PP2A regulatory subunit transcript, *Ppp2r3a*, in Rac1^cKI^ hearts at baseline and in response to hypertrophic stimulation, along with reduced protein levels and impaired localization of its gene products, PR72 and PR130. These data suggest *S*-acylated Rac1 may bolster compartmentalized cardiomyocyte PP2A function to prevent chronic overactivation of PKA in response to hypertrophic stress. Our results demonstrate an adaptive role for *S*-palmitoylation–dependent regulation of localized Rac1 activity in cardiomyocytes in vivo, which is essential for proper cardiac resilience against persistent PKA activation and heart failure in response to chronic hypertrophic stimulation.

## Results

### S-palmitoylation of Rac1 at Cys-178 regulates its activation.

**Figure 1:**
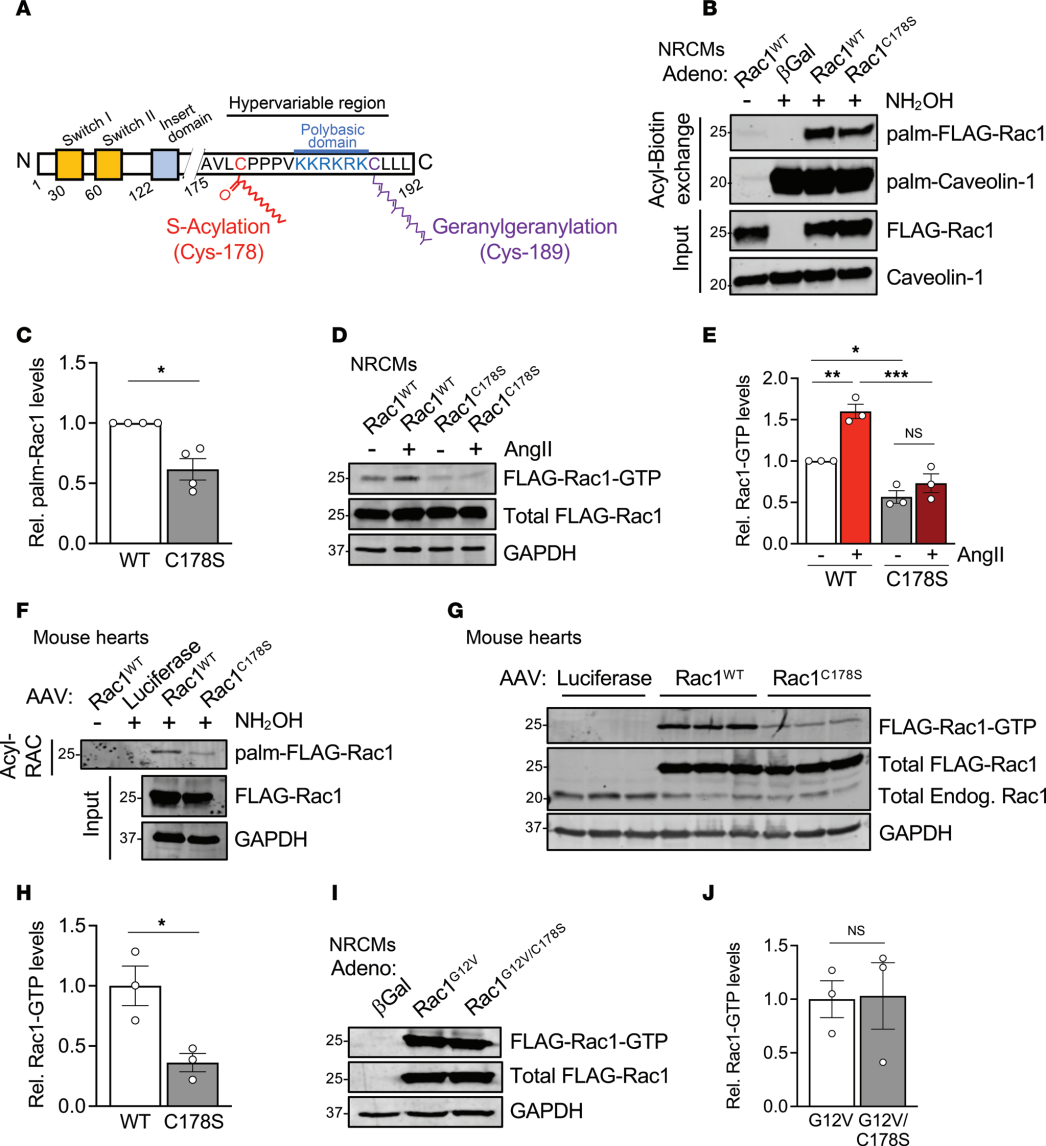
Loss of Cys-178 *S*-palmitoylation of Rac1 in cardiomyocytes reduces Rac1 activity. (**A**) A schematic of Rac1 protein, showing domain structure including the C-terminal hypervariable region with 3 membrane targeting motifs: the Cys-178 *S*-palmitoylation site (red), polybasic domain (blue), and C-terminal Cys-189 geranylgeranylation site (purple). (**B**) Immunoblotting and (**C**) quantification of *S*-acylated Rac1 affinity purified by acyl-biotin exchange from lysates of neonatal rat cardiomyocytes (NRCMs) transduced with adenoviruses encoding β-galactosidase (βGal, control), FLAG-Rac1^WT^, or FLAG-Rac1^C178S^. *n* = 4/group. (**D**) Immunoblotting and (**E**) quantification of active (GTP-bound) Rac1 affinity purified following pull-down with the p21-activated kinase p21-binding domain (PAK-PBD) in lysates of NRCMs overexpressing Rac1^WT^ or Rac1^C178S^ and treated with saline or angiotensin II (AngII, 10 μM, 48 hours). *n* = 3/group. (**F**) Immunoblotting for *S*-acylated Rac1 affinity purified by acyl resin–assisted capture (Acyl-RAC) and (**G**) active, GTP-bound Rac1 affinity purified by PAK-PBD pull-down assays from hearts of mice injected with AAV9s on postnatal day 6 to express N-terminally FLAG-tagged Rac1^WT^ or Rac1^C178S^ in cardiomyocytes in vivo and harvested at 4 months of age. (**H**) Quantification of GTP-bound Rac1 in the indicated hearts of AAV-injected mice. *n* = 3/group. (**I**) Immunoblotting and (**J**) quantification of Rac1-GTP purified from NRCMs transduced with adenoviruses encoding βGal (control) or N-terminally FLAG-tagged Rac1^G12V^ or Rac1^G12V/C178S^. *n* = 3/group. Relative *S*-acylated or active Rac1 levels were normalized to total input Rac1 levels in panels **C**, **E**, **H**, and **J**. *P < 0.05; **P < 0.01; ***P < 0.001 by Mann-Whitney test (**C**), 2-way ANOVA with post hoc Tukey’s multiple-comparison test (**E**), or unpaired, 2-tailed *t* test (**H** and **J**). NS, not significant.

Rac1 is permanently modified on its C-terminus at Cys-189 by the 20-carbon geranylgeranyl isoprenoid that is required for its intracellular trafficking and function as with other Rho family small GTPases (41–43) but can also be reversibly *S*-palmitoylated at Cys-178 (25, 33) (Figure 1A) to impart dynamic targeting to intracellular membranes. Previous literature has implicated Cys-178 as the major Rac1 palmitoylation site and shown that mutation of this cysteine residue to serine reduces steady-state GTP loading in immortalized cells (25). To determine whether Cys-178 of Rac1 is regulated by *S*-palmitoylation in cardiomyocytes, we overexpressed N-terminally FLAG-tagged wild-type Rac1 (Rac1^WT^) or *S*-palmitoylation–resistant Rac1^C178S^ in neonatal rat cardiomyocytes (NRCMs). We then assessed *S*-acylated protein levels using acyl-biotin exchange (ABE) (33, 44, 45) or acyl resin–assisted capture (Acyl-RAC) (33, 44, 46, 47). To evaluate Rac1 activity, we performed pull-downs with a GST fusion protein of the p21-binding domain (PBD) of the Rac1 effector, Pak1, that specifically binds GTP-loaded Rac1, allowing for the affinity purification of active Rac1 (25, 26, 33). As expected, expression of the Rac1^C178S^ mutant indeed resulted in less steady-state *S*-palmitoylation compared with Rac1^WT^ (Figure 1, B and C). Notably, this deficit in *S*-palmitoylation of the Rac1^C178S^ mutant was associated with reduced basal GTP loading and blunted activation following acute treatment with AngII (Figure 1, D and E). The intracellular membrane topography of adult cardiomyocytes is structurally unique from immature NRCMs, so we next assessed whether *S*-palmitoylation of Rac1 is required for its activity in the adult mouse heart. We injected WT mice on postnatal day 6 with adeno-associated virus serotype 9 (AAV9) to overexpress Rac1^WT^ or Rac1^C178S^ under the control of the cardiomyocyte-specific troponin T promoter and found a substantial reduction in *S*-palmitoylation (Figure 1F) and GTP loading of Rac1^C178S^ compared with Rac1^WT^ in the adult mouse heart (Figure 1, G and H), indicating modification of at least a subset of Rac1 protein at Cys-178 is essential for signaling activity in cardiomyocytes in vivo.

To determine whether Rac1 *S*-palmitoylation reduces Rac1 activity or is absolutely required for GTP loading of Rac1 in cardiomyocytes, we generated recombinant adenovirus to express Rac1 with a constitutively activating G12V mutation that impairs GTP hydrolysis with and without the C178S mutation of the *S*-palmitoylation site. NRCMs expressing either Rac1^G12V^ or the Rac1^G12V/C178S^ compound mutant exhibited robust GTP loading and activation of Rac1 (Figure 1, I and J), indicating that genetic abrogation of Rac1 *S*-palmitoylation via mutation of Cys-178 does not impair Rac1 protein folding or its ability to be GTP loaded in cardiomyocytes but rather impacts steady-state levels of active Rac1, likely through regulating the kinetics of targeting of Rac1 to cardiomyocyte intracellular membranes accessible to guanine nucleotide exchange factors (GEFs) that enzymatically load Rac1 with GTP.

### Generation and characterization of cardiomyocyte-specific Rac1 C178S–knockin mice.

**Figure 2:**
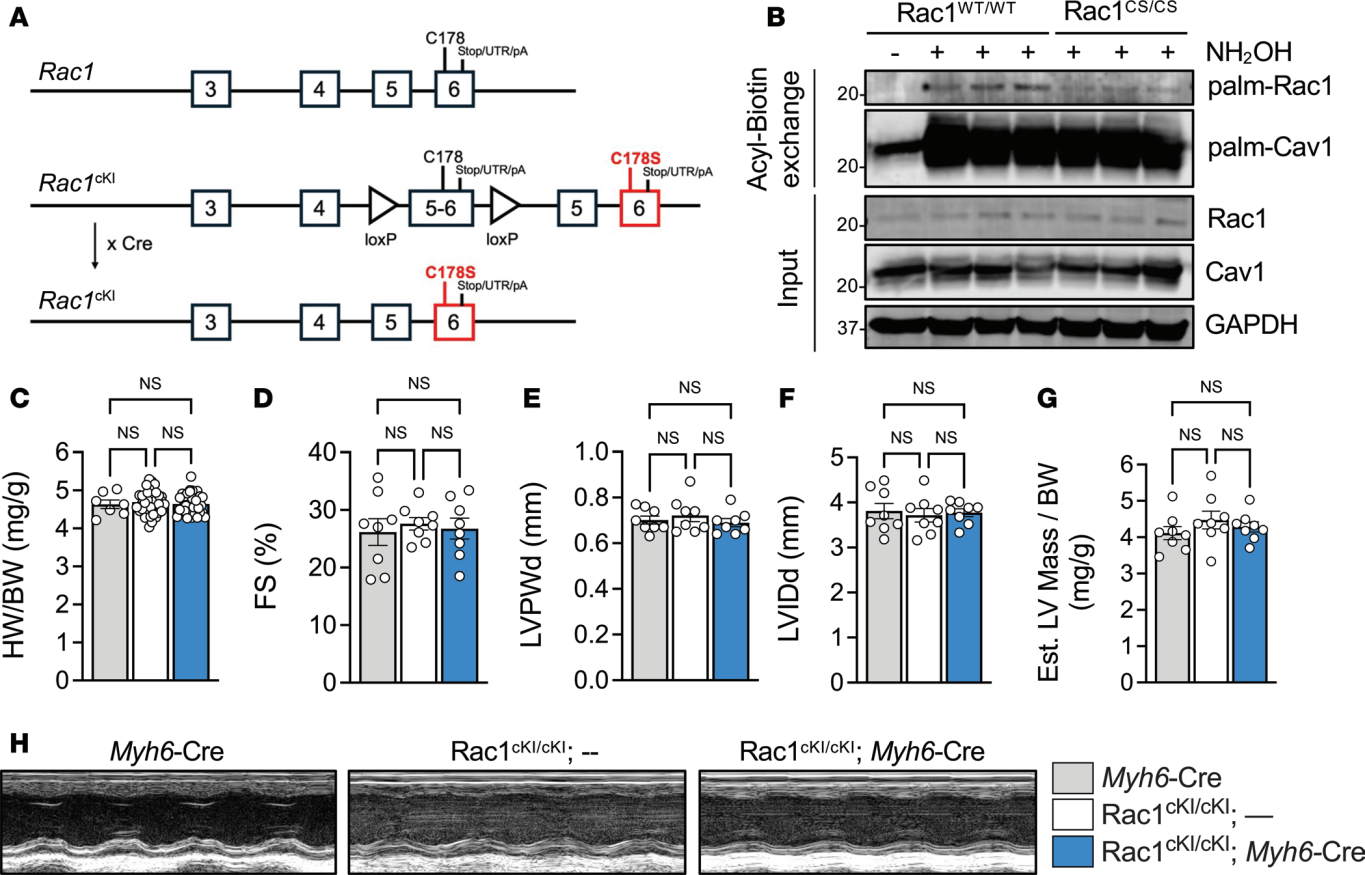
Generation and characterization of Rac1 conditional knockin mice. (**A**) Schematic for generation of a targeted *Rac1* allele with conditional knockin mutation encoding C178S (*Rac1*^cKI^). A fused WT exon 5/6 is flanked by *loxP* sites, followed by WT exon 5 and exon 6 encoding a C178S mutation for expression of WT Rac1 protein in the absence of Cre recombinase but Rac1^C178S^ in the presence of Cre. Cre-mediated recombination to excise the *loxP*-targeted WT exon 5/6 results in the *Rac1* locus encoding Rac1 protein with the C178S mutation. Crossing *Rac1*^cKI^ with *Myh6*-Cre mice drives recombination and expression of the Rac1^C178S^ mutant specifically in cardiomyocytes. UTR, untranslated region; pA, polyA tail. (**B**) Immunoblotting of *S*-acylated proteins purified by acyl-biotin exchange from cardiac lysates of control or whole-body Rac1-knockin mice. *S*-acylation of caveolin-1 was used as a positive control. (**C**) Heart weight–to–body weight (HW/BW) ratios of the indicated genotypes of mice at 8–10 weeks of age. Echocardiographic assessment of (**D**) fractional shortening (FS), (**E**) left ventricular posterior wall thickness at diastole (LVPWd), (**F**) left ventricular inner diameter at diastole (LVIDd), and (**G**) estimated left ventricular mass–to–body weight ratio at 4 months of age in *Myh6*-Cre alone (gray), Rac1^cKI/cKI^ (white), and Rac1^cKI/cKI^; *Myh6*-Cre (blue) mice. 1-way ANOVA with post hoc Tukey’s multiple-comparison test. NS, not significant. *n* = 6–36/group for **C**, *n* = 8/group for **D**–**G**. (**H**) Representative M-mode images of the indicated genotypes at 4 months of age.

To better assess the function of Rac1 palmitoylation in response to hypertrophic stimuli without artifacts of Rac1 overexpression, we generated conditional knockin mice to elicit Cre-dependent expression of palmitoylation-resistant Rac1^C178S^ at endogenous levels (Figure 2A). Despite global deletion of Rac1 being embryonic lethal (48), global loss of Rac1 *S*-palmitoylation at Cys-178 in whole-body Rac1-knockin mice, which were generated by crossing Rac1^cKI^ mice with a ubiquitously Cre-expressing line, did not result in obvious phenotypic abnormalities or any cardiac phenotype up to 4 months of age and genotypes from heterozygous crosses were produced at expected Mendelian ratios (Supplemental Figure 1, A–D; supplemental material available online with this article; https://doi.org/10.1172/jci.insight.193733DS1). Hearts collected from whole-body knockin mice exhibited reduced steady-state Rac1 *S*-palmitoylation compared with littermate controls, as expected (Figure 2B).

To specifically assess the role of Rac1 *S*-palmitoylation in cardiomyocytes, we generated cardiomyocyte-specific knockin mice under the control of a Cre recombinase driven by the α-myosin heavy chain (αMHC/*Myh6*) promoter. Cardiomyocyte-specific Rac1^cKI^ mice did not develop cardiac hypertrophy in the absence of stimulus (Figure 2C) and had no significant changes in cardiac structure or function compared with control genotypes as assessed by echocardiography up to 4 months of age (Figure 2, D–H, and Supplemental Table 1), suggesting modification of Rac1 at Cys-178 is largely dispensable for basal cardiac function.

As *S*-palmitoylation is often a regulator of compartmentalization at intracellular membranes, we further assessed the impact of Rac1 modification at Cys-178 on its localization in cardiomyocytes. We did not observe marked differences in the localization of the Rac1^C178S^ mutant protein compared to Rac1^WT^ by immunocytochemistry in adult cardiomyocytes isolated from Rac1^cKI^ hearts (Supplemental Figure 2, A and B) nor was partitioning of Rac1^C178S^ into lipid rafts altered compared to Rac1^WT^, as detected by biochemical fractionation and immunoblotting of whole-body Rac1-knockin hearts (Supplemental Figure 3, A–D), suggesting *S*-palmitoylation of Rac1 more subtly alters Rac1 targeting to cardiomyocyte membrane microdomains. We did, however, observe a modest but significant increased presence of the Rac1^C178S^ palmitoylation-resistant mutant in the cytoplasmic fraction at the expense of reduced levels in the membrane-enriched (1% Triton X-100 soluble) fraction (Supplemental Figure 3, E–H), suggesting reduced association of Rac1 with intracellular cardiomyocyte membranes in the absence of *S*-palmitoylation. In vivo proximity labeling proteomics with cardiomyocyte-specific AAV9-mediated overexpression of BioID2 fusion proteins of Rac1^WT^ or Rac1^C178S^ for purification of proteins within an approximately 10–20 nm radius (49) revealed a similar close-proximity proteome for the Rac1^C178S^ mutant and Rac1^WT^ (Supplemental Figure 4, A–I), suggesting Rac1 largely associates with the same intracellular compartments in the absence of *S*-palmitoylation and suggesting *S*-palmitoylation cycling of Rac1 modulates the kinetics of Rac1 localization and signaling activity at cardiomyocyte membranes or deploys a portion of Rac1 for targeting to discrete intracellular membranes for GEF-mediated activation.

### Loss of Rac1 S-palmitoylation at Cys-178 results in more severe cardiac hypertrophy and functional decompensation in response to AngII infusion or pressure overload.

**Figure 3:**
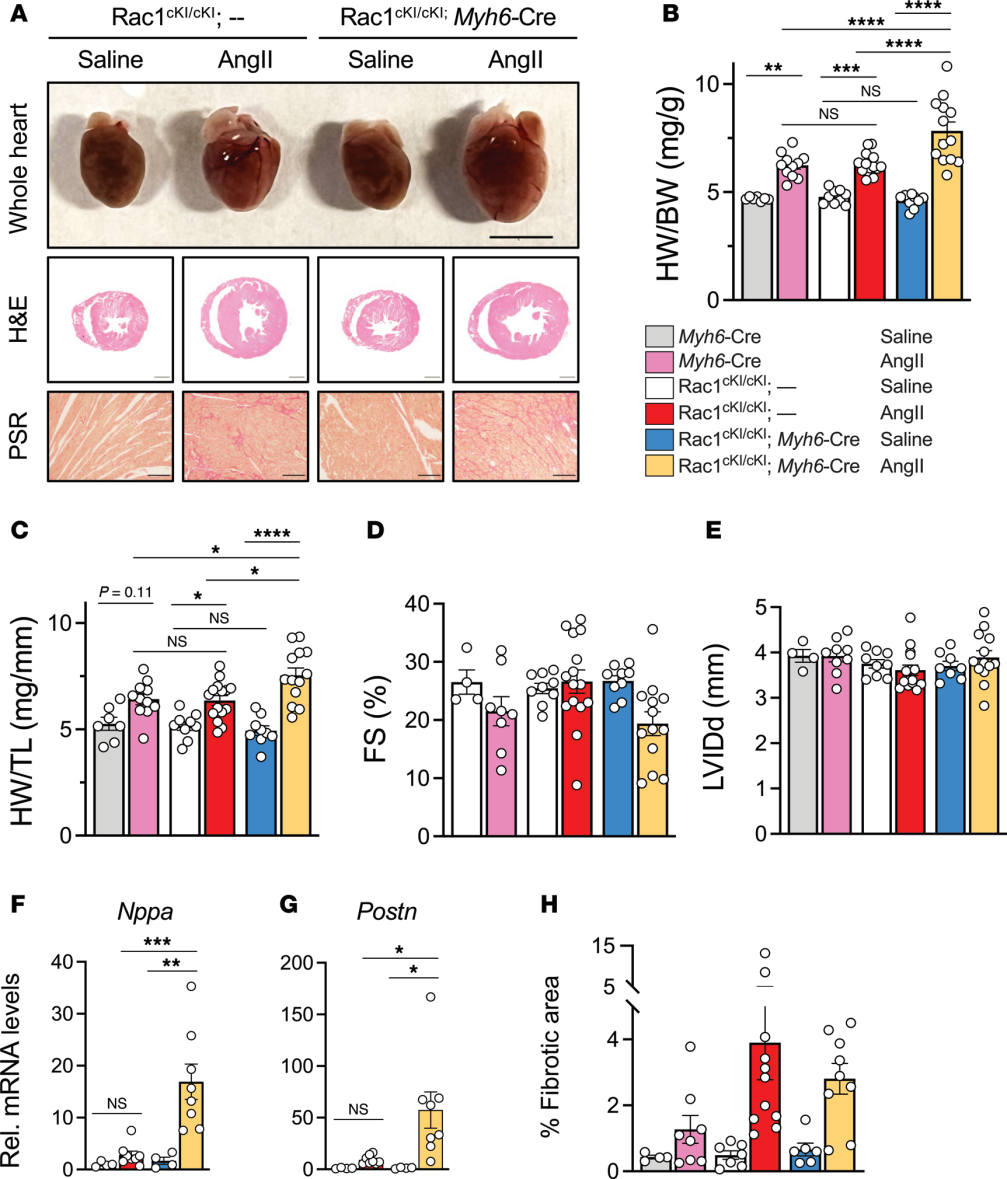
Cardiomyocyte-specific Rac1 conditional knockin mice develop more severe cardiac hypertrophy in response to AngII. (**A**) Representative images of whole hearts and H&E- and Picrosirius red–stained (PSR-stained) cardiac sections from control and Rac1 cardiomyocyte-specific knockin mice infused with saline or AngII (3 mg/kg/d) for 14 days. Scale bars: 5 mm (whole hearts), 1 mm (H&E), 100 μm (PSR). (**B**) Heart weight–to–body weight (HW/BW) and (**C**) heart weight–to–tibia length (HW/TL) ratios of *Myh6*-Cre alone, Rac1^cKI/cKI^, and Rac1^cKI/cKI^; *Myh6*-Cre mice treated with saline or AngII for 14 days. Echocardiographic assessment of (**D**) fractional shortening (FS) and (**E**) left ventricular inner diameter at diastole (LVIDd) following 2 weeks of saline or AngII infusion. Quantification of (**F**) *Nppa* and (**G**) *Postn* transcript levels by qPCR in mouse hearts of the indicated genotype and treatment. (**H**) Quantification of fibrotic area from PSR-stained sections of saline or AngII-treated control and Rac1^cKI^ mice. *P < 0.05; **P < 0.01; ***P < 0.001; ****P < 0.0001 by 2-way ANOVA with post hoc Tukey’s multiple-comparison test. NS, not significant. *n* = 7–15 per group for **B** and **C**, *n* = 4–15 per group for **D** and **E**, *n* = 4–8 per group for **F** and **G**, *n* = 4–11 per group for **H**.

To investigate the role of Rac1 *S*-palmitoylation in a mouse model of pathologic cardiac hypertrophy, we implanted osmotic minipumps to chronically infuse either saline or AngII (3 mg/kg/day) for 2 weeks. Control mice (either *Myh6*-Cre alone or Rac1^cKI/cKI^ mice without *Myh6*-Cre) consistently displayed modest increases in cardiac hypertrophy in response to AngII, as expected (Figure 3, A–C). However, cardiomyocyte-specific Rac1 knockin mice (Rac1^cKI/cKI^; *Myh6*-Cre) exhibited significantly exacerbated cardiac hypertrophy in response to AngII (Figure 3, A–C), indicating hypersensitivity to AngII-induced cardiac hypertrophy in the absence of Rac1 *S*-palmitoylation. No statistically significant differences in left ventricular structure or function were observed between genotypes in response to AngII as assessed by echocardiography, although Rac1^cKI^ mice exhibited a trend toward reduced systolic function (reduced fractional shortening) in response to AngII that was not observed with other genotypes (Figure 3, D and E, and Supplemental Table 2). Consistent with increased cardiac hypertrophy, Rac1^cKI^ mice treated with AngII also had elevated transcript levels of the pathological cardiac stress marker gene *Nppa* (Figure 3F) and fibrotic marker gene *Postn* (Figure 3G). Similar trends were observed for other hypertrophic and fibrotic transcripts (Supplemental Figure 5A); however, there were no significant differences in interstitial fibrosis between genotypes as assessed by Picrosirius red (PSR) staining (Figure 3, A and H). Assessment of myocardial oxidative stress by dihydroethidium staining of cardiac sections revealed no changes in the basal or AngII-stimulated superoxide levels in Rac1^cKI^ hearts (Supplemental Figure 6, A–D). This finding is consistent with the lack of a cardioprotective phenotype and suggests *S*-palmitoylation of Rac1 does not appreciably regulate NADPH oxidase 2 (NOX2) activity in cardiomyocytes.

**Figure 4:**
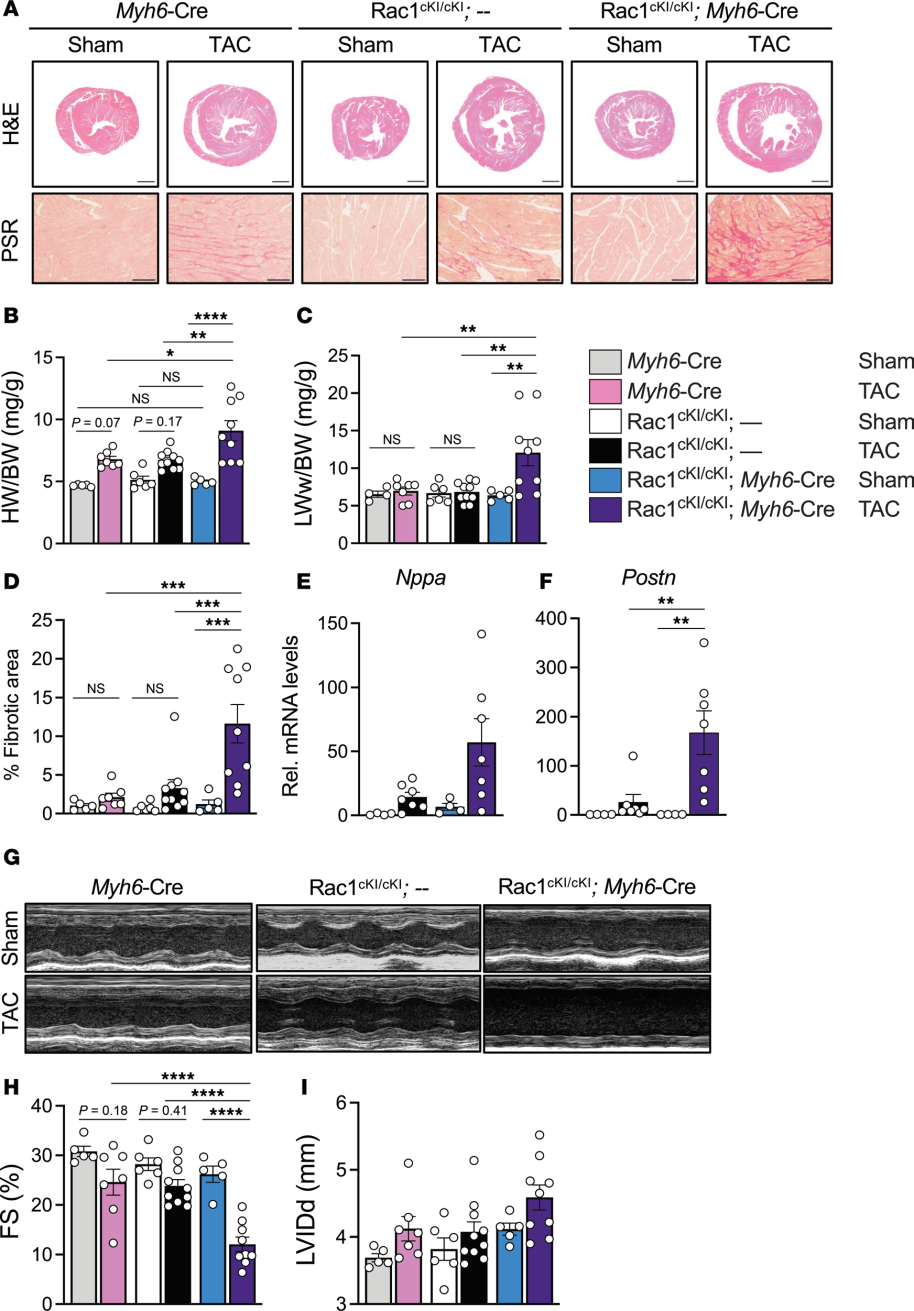
Cardiomyocyte-specific Rac1 conditional knockin mice develop more severe cardiac hypertrophy and functional decompensation following left ventricular pressure overload. (**A**) Representative H&E- and Picrosirius red–stained (PSR-stained) cardiac sections of control and Rac1 cardiomyocyte-specific knockin mice 8 weeks after sham or transverse aortic constriction (TAC) surgery. Scale bars: 1 mm (H&E) and 100 μm (PSR). (**B**) Heart weight–to–body weight (HW/BW) ratios, (**C**) wet lung weight–to–body weight (LWw/BW) ratios, and (**D**) quantification of cardiac interstitial fibrosis from PSR-stained sections in sham- or TAC-operated mice of the indicated genotypes 8 weeks after surgery. Quantification of (**E**) *Nppa* and (**F**) *Postn* transcript levels by qPCR in mouse hearts of sham or TAC-operated control or Rac1^cKI^ mice 8 weeks after surgery. (**G**) Representative M-mode echocardiograms from sham- or TAC-operated control or Rac1^cKI^ mice 8 weeks after surgery and quantification of (**H**) fractional shortening (FS) and (**I**) left ventricular inner diameter at diastole (LVIDd). *P < 0.05; **P < 0.01; ***P < 0.001; ****P < 0.0001 by 2-way ANOVA with post hoc Tukey’s multiple-comparison test. NS, not significant. *n* = 5–10 per group for **B**, **D**, **H**, and **I**, *n* = 4–10 per group for **C**, *n* = 4–7 per group for **E** and **F**.

To investigate the role of Rac1 *S*-palmitoylation in cardiomyocytes in response to another chronic model of cardiac hypertrophy induced by left ventricular overload and mechanical stress, we subjected 8- to 10-week-old Rac1^cKI^ mice to TAC, followed by assessment of cardiac functional and structural changes by echocardiography, histopathology, and molecular analyses. After 8 weeks of pressure overload, we observed increased cardiac hypertrophy (Figure 4, A and B), pulmonary edema (Figure 4C), and interstitial cardiac fibrosis (Figure 4, A and D) in TAC-operated Rac1^cKI^ mice compared with TAC-operated control mice, indicating more severe cardiac disease and heart failure in the absence of Rac1 *S*-palmitoylation in response to long-term pressure overload. Rac1^cKI^ hearts exhibited higher transcript levels of the cardiac stress marker genes *Nppa*, *Nppb*, and *Myh7* and profibrotic genes *Postn* and *Col1a1* compared with hearts of control mice subjected to TAC (Figure 4, E and F, and Supplemental Figure 5B). This is consistent with more severe cardiac hypertrophy (Figure 4, A and B) and fibrosis (Figure 4, A and D) and indicative of more adverse remodeling in response to chronic pressure overload. Moreover, echocardiography revealed much more severe systolic dysfunction in Rac1^cKI^ mice in response to pressure overload, with a significantly greater reduction in fractional shortening in response to TAC compared with control genotypes (Figure 4, G and H, and Supplemental Table 3) and a trend toward greater left ventricular dilation (Figure 4, G and I, and Supplemental Table 3), indicating loss of Rac1 *S*-palmitoylation is maladaptive and elicits functional decompensation in the pressure-overloaded heart.

### Loss of Rac1 S-palmitoylation at Cys-178 results in more severe cardiac hypertrophy and functional decompensation in response to chronic cardiomyocyte AT1R signaling.

**Figure 5:**
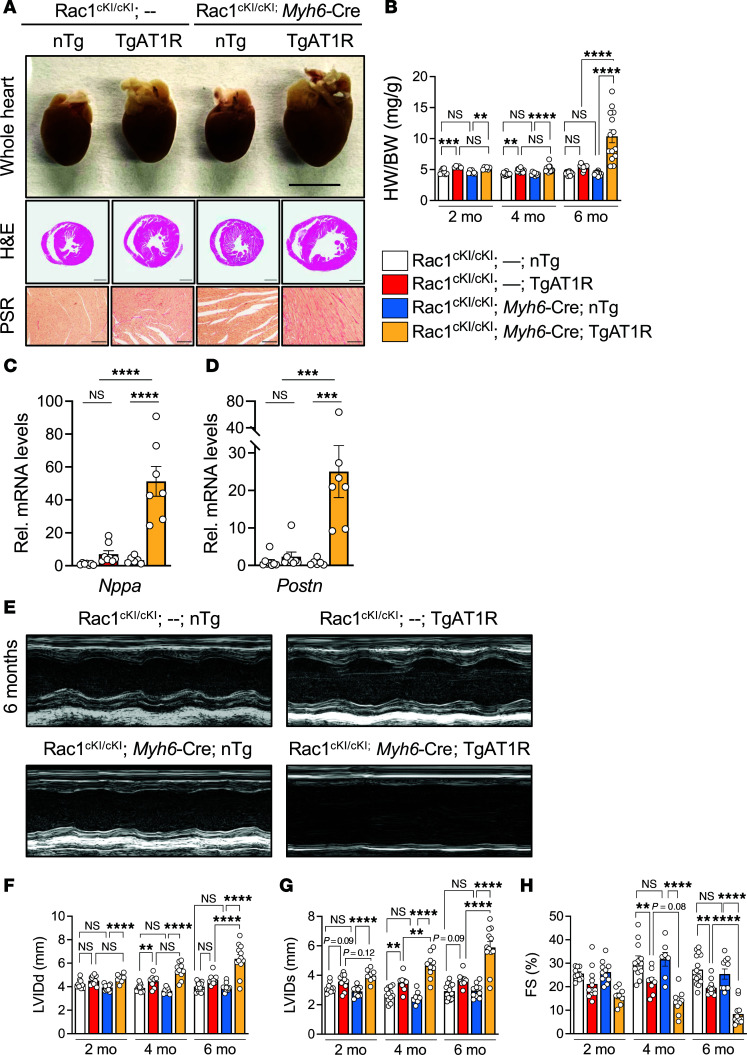
Cardiomyocyte-specific Rac1 conditional knockin mice develop more severe cardiac hypertrophy and functional decompensation in response to transgenic AT1R overexpression. (**A**) Representative images of whole hearts and H&E- and Picrosirius red–stained (PSR-stained) cardiac sections of control and Rac1 cardiomyocyte-specific knockin mice with or without transgenic AT1R overexpression at 6 months of age. Scale bars: 5 mm (whole hearts), 1 mm (H&E), and 100 μm (PSR). (**B**) Heart weight–to–body weight (HW/BW) ratios at 2 months, 4 months, and 6 months of age. (**C**) *Nppa* and (**D**) *Postn* mRNA levels in hearts from 6-month-old mice of the indicated genotypes. (**E**) Representative M-mode echocardiograms at 6 months of age of Rac1^cKI^ mice with and without transgenic AT1R overexpression and quantification of (**F**) left ventricular inner diameter at diastole (LVIDd), (**G**) left ventricular inner diameter at systole (LVIDs), and (**H**) fractional shortening (FS) at 2, 4, and 6 months of age. **P < 0.01; ***P < 0.001; ****P < 0.0001 by 2-way ANOVA with post hoc Tukey’s multiple-comparison test. NS, not significant. *n* = 5–15 per group for **B**, *n* = 6–8 per group for **C** and **D**, *n* = 7–15 per group for **F**–**H**. nTg, non-transgenic.

To examine the role of Rac1 *S*-palmitoylation in a model of pathologic cardiac hypertrophy and failure driven by hypertrophic signaling in cardiomyocytes and independent of changes in blood pressure, we crossed Rac1^cKI^ mice with transgenic mice overexpressing AT1R in cardiomyocytes (TgAT1R mice) (10). In our hands, TgAT1R mice develop mild cardiac hypertrophy relative to non-transgenic littermates by 2 months and maintain a similar degree of hypertrophy, dysfunction, and cardiac pathology up to 6 months (Figure 5, A–H, and Supplemental Tables 4–6). Rac1^cKI^ mice overexpressing AT1R developed a similar degree of hypertrophy as controls overexpressing AT1R at 2 and 4 months of age but a substantial exacerbation of cardiac hypertrophy was observed in Rac1^cKI^ mice overexpressing AT1R at 6 months of age (Figure 5, A and B). Consistent with this, we observed further increases in cardiac stress marker genes *Nppa* and *Nppb* and fibrotic marker genes *Postn* and *Col1a1* in the hearts of Rac1^cKI^ mice expressing the AT1R transgene compared with AT1R-transgenic mice expressing Rac1^WT^ (Figure 5, C and D, and Supplemental Figure 5C). Echocardiography analyses revealed more severe left ventricular dilation and systolic dysfunction in Rac1^cKI^ mice with transgenic AT1R expression as early as 2 months that continues to progress at 4 and 6 months of age, highlighting a significantly more severe dilated cardiomyopathy phenotype in Rac1^cKI^ mice with transgenic AT1R expression compared with mice with AT1R overexpression on its own (Figure 5, E–H, and Supplemental Tables 4–6).

### Rac1 S-palmitoylation restrains hyperphosphorylation of PKA substrates in response to adrenergic stimulation and chronic hypertrophic stress.

**Figure 6:**
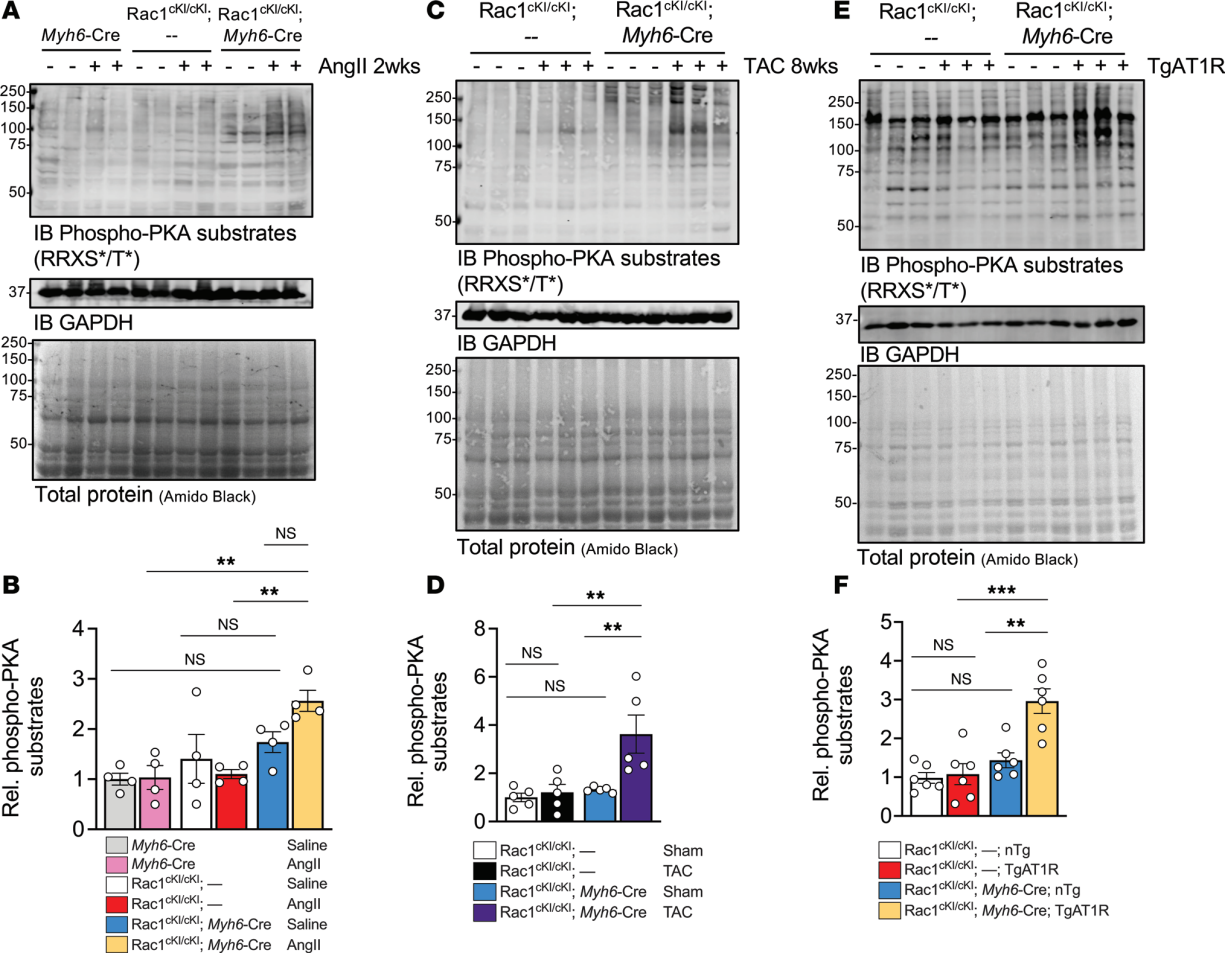
Rac1 conditional knockin mice subjected to hypertrophic stress have elevated myocardial PKA substrate phosphorylation. Representative immunoblotting and quantification, respectively, of phosphorylated PKA substrates in hearts from mice of the indicated genotypes (**A** and **B**) infused with saline or AngII for 2 weeks, (**C** and **D**), 8 weeks after sham or TAC surgeries, and (**E** and **F**) at 4 months of age with or without transgenic AT1R expression. GAPDH and amido black total protein stain were used as loading controls. Amido black intensity was used for normalization of phosphorylated PKA substrate quantification in **B**, **D**, and **F**. **P < 0.01; ***P < 0.001 by 2-way ANOVA with post hoc Tukey’s multiple-comparison test. NS, not significant. *n* = 4 per group for **B**, *n* = 5 per group for **D**, *n* = 6 per group for **F**.

PKA has indispensable roles in phosphorylating excitation-contraction (E-C) coupling machinery to mediate enhanced contractility in response to β-adrenergic stimulation and in promoting pathologic cardiac hypertrophy in response to pressure overload (16, 20). To probe the mechanisms underlying the unexpectedly maladaptive phenotype resulting from genetic ablation of Rac1 Cys-178 *S*-palmitoylation in the response of the heart to pathologic stress, we examined regulation of PKA activity in Rac1^cKI^ hearts in response to chronic pathological hypertrophy models by immunoblotting for phosphorylated PKA substrates (using an antibody targeting the immunoreactive RRXS*/T* phosphorylation motif). Strikingly, after 2 weeks of AngII infusion, Rac1^cKI^ hearts exhibited a significant increase in phosphorylation of PKA substrates compared with control hearts (Figure 6, A and B). A recent study found cardiac PKA activity to be elevated within hours to days after TAC but PKA activity returned to sham levels after 2 weeks (20). We probed PKA substrate phosphorylation in hearts in response to 8 weeks of TAC and observed normal levels in control hearts but an accumulation of phosphorylated PKA substrates in Rac1^cKI^ hearts (Figure 6, C and D), suggesting Rac1 *S*-palmitoylation cycling may be necessary to repress sustained PKA activity or to promote dephosphorylation of PKA substrates. Moreover, a significant elevation in phosphorylation of PKA substrates was also found in Rac1^cKI^ hearts at 4 months of age with cardiomyocyte AT1R overexpression (Figure 6, E and F), suggesting Rac1 *S*-palmitoylation is required to prevent sustained PKA activity or by some other mechanism to mitigate hyperphosphorylation of PKA substrates in response to chronic hypertrophic stress.

**Figure 7:**
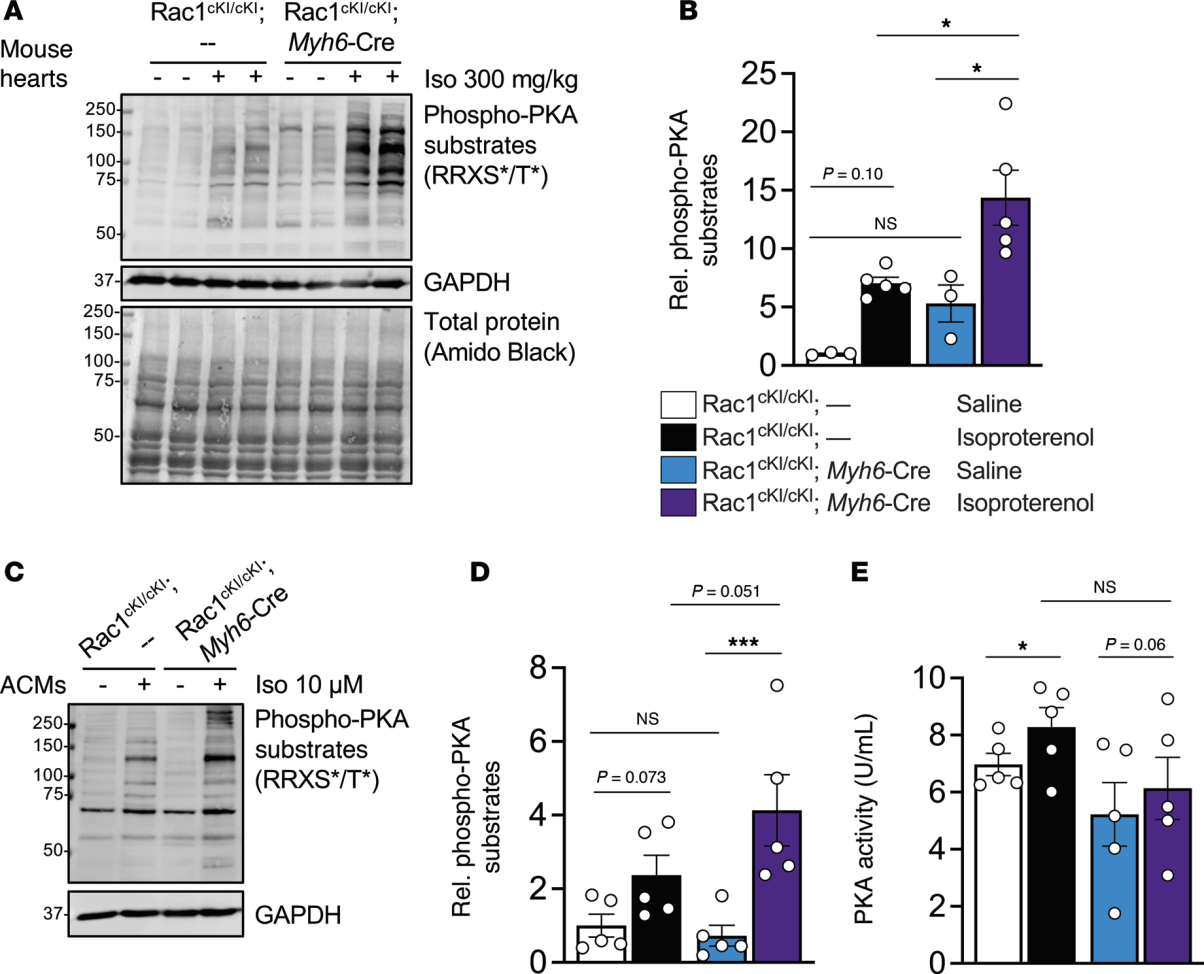
Rac1 conditional knockin cardiomyocytes exhibit hyperphosphorylation of PKA substrates in response to acute β-adrenergic stimulation. (**A**) Immunoblotting and (**B**) quantification of phosphorylated PKA substrates in heart lysates from the indicated genotypes of mice 10 minutes after being injected with saline or 300 mg/kg isoproterenol. GAPDH and amido black total protein stain were used as loading controls. *P < 0.05 by 2-way ANOVA with post hoc Tukey’s multiple-comparison test. *n* = 3–5/group. (**C**) Immunoblotting and (**D**) quantification of phosphorylated PKA substrates in isolated adult cardiomyocytes treated with saline or 10 μM isoproterenol for 5 minutes. GAPDH was used for normalization of phosphorylated PKA substrate levels in **B** and **D**. (**E**) PKA enzymatic activity in saline or isoproterenol-treated adult cardiomyocytes from control or Rac1^cKI^ mice. Cardiomyocytes isolated from the same mice were used for saline and isoproterenol treatments in **C**–**E**. *P < 0.05; ***P < 0.001 by 2-way repeated measures ANOVA with post hoc Tukey’s multiple-comparison test. *n* = 5/group in **D** and **E**. NS, not significant.

To assess whether *S*-palmitoylation of Rac1 at Cys-178 has an acute role in modulating PKA substrate phosphorylation in cardiomyocytes, we injected Rac1^cKI^ mice with the β-adrenergic agonist isoproterenol (300 mg/kg) and evaluated phosphorylation of PKA substrates in the heart 10 minutes after injection. Rac1^cKI^ hearts exhibited a substantially greater increase in phosphorylation of PKA substrates in response to isoproterenol compared with control genotypes (Figure 7, A and B), suggesting a role for Rac1 *S*-palmitoylation in acutely sensing and preventing excessive phosphorylation of the PKA phosphoproteome in response to β-adrenergic stimulation in vivo. Furthermore, adult mouse cardiomyocytes (AMCMs) isolated from Rac1^cKI^ mice treated with isoproterenol in vitro for 5 minutes similarly showed substantially greater enhancement of β-adrenergic–stimulated phosphorylation of PKA substrates (Figure 7, C and D). Isoproterenol-induced PKA enzymatic activity was not altered between cardiomyocytes isolated from control and Rac1^cKI^ hearts (Figure 7E), suggesting hyperphosphorylation of PKA substrates associated with loss of Rac1 *S*-palmitoylation at Cys-178 is not due to increases in total cellular PKA activity.

### Rac1^cKI^ hearts downregulate the Ppp2r3a regulatory subunit of PP2A.

**Figure 8:**
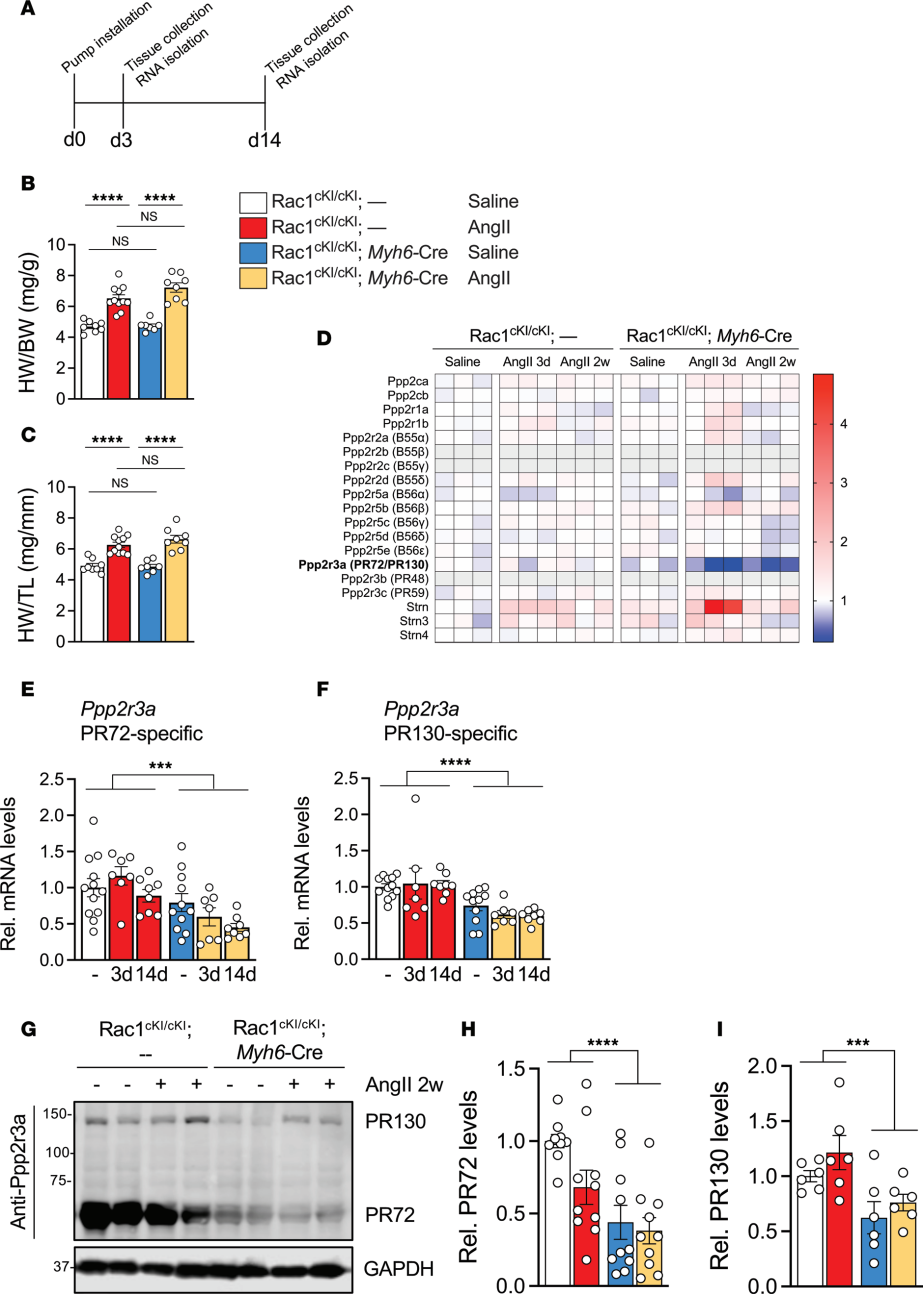
*Ppp2r3a*/PR72 levels are reduced in Rac1 conditional knockin mouse hearts. (**A**) Schematic for RNA-seq analyses. Hearts were collected for RNA isolation at 3 days and 14 days after osmotic pump installation. (**B**) Heart weight–to–body weight (HW/BW) ratios and (**C**) heart weight–to–tibia length (HW/TL) ratios of mice treated with saline or AngII (3 mg/kg/d) for 3 days. *n* = 7–11 mice/group. (**D**) Heatmap of all PP2A catalytic, regulatory, and scaffolding subunit transcript levels from bulk RNA-seq. Deeper blue shades indicate reduced expression and deeper red shades indicate increased expression. Gray boxes indicate low abundance transcripts with FPKM levels <1. (**E**) PR72 and (**F**) PR130 *Ppp2r3a* splice variant mRNA levels quantified by qPCR in hearts from mice treated with saline or AngII for 3 or 14 days. *n* = 7–11/group. (**G**) Representative immunoblotting for PR72 and PR130 in hearts from mice of the indicated genotypes treated with saline or AngII for 14 days. GAPDH was used as a loading control. (**H**) PR72 and (**I**) PR130 protein levels relative to GAPDH in hearts of control or Rac1^cKI^ mice treated with saline or AngII for 14 days. ***P < 0.001; ****P < 0.0001 by 2-way ANOVA with post hoc Tukey’s multiple-comparisons test. NS, not significant.

To further probe molecular mechanisms potentially underlying hypersensitivity of Rac1^cKI^ myocytes to hyperactive PKA signaling, we performed bulk RNA-seq on hearts of Rac1^cKI^ and control mice infused with saline or AngII for 3 days (Figure 8A), prior to significant genotypic differences in cardiac hypertrophy (Figure 8, B and C), or for 14 days when Rac1^cKI^ mice have significantly more cardiac hypertrophy in response to AngII (Figure 3, A–C, and Figure 8A). Pathway analyses were performed on differentially expressed genes identified between control and Rac1^cKI^ hearts treated with saline or AngII for 3 days (Supplemental Figure 7, A–G). There was an enrichment of differentially expressed genes related to cardiac muscle contraction in saline-treated Rac1^cKI^ hearts compared with controls (Supplemental Figure 7F). We found a striking downregulation of transcript levels of *Ppp2r3a*, encoding the PR72/PR130 B family regulatory subunit of PP2A, in Rac1^cKI^ hearts at baseline and further reduced *Ppp2r3a* mRNA levels following 3 or 14 days of AngII infusion (Figure 8D). PR72-containing PP2A trimers regulate cardiomyocyte E-C coupling in large part via the regulated dephosphorylation of myofilament and sarcoplasmic reticulum (SR) calcium cycling substrates of PKA (22, 39, 40), providing a potential mechanistic link to the observed hyperphosphorylation of PKA substrates in cardiomyocytes resistant to Rac1 *S*-palmitoylation (Figure 6, A–F, and Figure 7, A–D). *Ppp2r3a* is alternatively spliced to form 2 primary gene products that function as PP2A phosphatase regulatory subunits, the cardiac-enriched PR72 isoform and PR130 (50). We confirmed downregulation of *Ppp2r3a* transcript levels in hearts of Rac1^cKI^ mice by qPCR using primers that specifically recognize PR72 and PR130 transcript variant mRNAs (Figure 8, E and F) and Western blotting using an antibody that recognizes both isoforms revealed reduced PR72 and PR130 protein levels in Rac1^cKI^ hearts at baseline and in response to AngII (Figure 8, G–I). Furthermore, while PR72 is reliably localized to the M-line in control cardiomyocytes, its distribution in Rac1^cKI^ cardiomyocytes, although not completely absent from the M-line, is more diffuse, suggesting PR72 is improperly localized in the absence of Rac1 *S*-palmitoylation (Supplemental Figure 8, A and B). These data suggest that loss of Rac1 *S*-palmitoylation at Cys-178 may render cardiomyocytes hypersensitive to PKA-mediated phosphorylation due to impaired PR72-regulated PP2A activity.

## Discussion

While the identification of intracellular signaling mechanisms that provoke cardiac hypertrophy and adverse myocardial remodeling has aided and enhanced our understanding of this complex process, there remain gaps in knowledge in our understanding of how the heart adapts to chronic stress and a dire need to uncover novel pathways that promote resiliency to cardiac maladaptation and heart failure. *S*-palmitoylation is a reversible posttranslational modification of cysteines with saturated fatty acids that modulates protein membrane targeting, intracellular trafficking, stability, membrane topography, and/or interactions with cofactors (8, 35, 42). In the case of soluble cytosolic proteins such as small GTPases that function as molecular switches to transduce signals from intracellular membranes, *S*-palmitoylation can impart dynamic control necessary for proper spatiotemporal activation of signal transduction circuitry downstream of plasmalemmal receptors (8, 25, 33, 46, 51, 52). In this study, we elucidated what we believe is a novel role for *S*-palmitoylation of Rac1 in the regulation of its cardiomyocyte signaling activity, β-adrenergic responsiveness, and propensity to heart failure in response to pathologic cardiac stress.

Despite global Rac1 deletion being embryonically lethal (48), global loss of Rac1 *S*-palmitoylation at Cys-178 in whole-body knockin mice does not result in gross developmental abnormalities or mortality, and cardiomyocyte-specific Rac1 conditional knockin mice do not display changes in cardiac structure or function with age, suggesting Rac1 *S*-palmitoylation at Cys-178 is dispensable for proper development, postnatal growth, and cardiac homeostasis. Cardiomyocyte-specific loss of Rac1 in mice is protective against AngII- (26) or pressure overload–induced (30) cardiac hypertrophy and the development of diabetic cardiomyopathy (53), suggesting Rac1 signaling induced by hypertrophic stimuli is pathological. In contrast, Rac1^cKI^ mice expressing palmitoylation-resistant Rac1^C178S^ solely in cardiomyocytes develop more severe cardiac disease in response to pharmacological and genetic models of hypertrophic stress, including AngII infusion, pressure overload, and cardiomyocyte-specific overexpression of AT1R that is associated with enhanced myocardial phosphorylation of PKA targets in all cases, indicating unexpected adaptive roles for signaling by *S*-palmitoylated Rac1 in restraining cardiac hypertrophy, maladaptation, and hyperactivation of the PKA-dependent phosphoproteome in response to stress.

We found that Cys-178 is a major site of Rac1 regulation by *S*-palmitoylation necessary for its GTP loading and activation, likely through targeting Rac1 to specific sarcolemmal membrane microdomains containing or accessible to a GEF, and that abrogation of Rac1 *S*-palmitoylation impairs cardiomyocyte stress signaling. *S*-palmitoylation of Rac1 at Cys-178 was previously demonstrated to be essential for its activation, partitioning into lipid rafts, and remodeling of the actin cytoskeleton in immortalized migratory cells (25). We did not observe consistent changes in Rac1 localization in adult cardiomyocytes with the C178S knockin mutation by static immunocytochemistry, which could be mainly due to insufficient sensitivity given the very low portion of total cellular Rac1 protein that is *S*-palmitoylated at steady state (<2%). However, biochemical fractionation of mouse hearts revealed increased extraction of palmitoylation-deficient Rac1^C178S^ in cytosol-enriched fractions and reduced extraction in membrane-enriched fractions, suggesting that loss of Rac1 *S*-palmitoylation reduces its association with the sarcolemma or other intracellular cardiomyocyte membranes. Indeed, proximity-labeling proteomics also revealed a largely similar interactome for Rac1^C178S^ and Rac1^WT^, suggesting Rac1 *S*-palmitoylation likely more subtly affects Rac1 targeting to specific membrane microdomains where it has access to distinct cardiomyocyte effectors that are not detectable by assays employed here.

It is also noteworthy that *S*-palmitoylation/depalmitoylation cycling of small GTPases and other soluble cytosolic signaling proteins can be necessary to mediate sustained intracellular signaling required to elicit changes in cellular physiology downstream of receptor sensing of extracellular stimuli. For example, *S*-palmitoylation cycling of signal transducer and activator of transcription 3 (STAT3) targets it to activated cytokine receptors for phosphorylation-dependent activation and ultimate nuclear translocation and transcriptional activity necessary for T helper 17 (Th17) cell differentiation and the pathogenesis of inflammatory bowel disease (51) and *S*-palmitoylation cycling of oncogenic mutant N-Ras is required for its ability to promote myeloid cell transformation and leukemia (52, 54, 55). Loss of Rac1 *S*-palmitoylation by genetic mutation of the site does not completely eliminate its ability to be activated in cardiomyocytes, as expression of a Rac1 protein with the C178S mutation along with a constitutively active G12V mutation that prevents GTP hydrolysis results in robust GTP loading similar to the G12V mutation alone. Thus, *S*-palmitoylation of Rac1 is poised to deploy nanoscale targeting of intracellular Rac1 GTPase activity in response to stress signals, mediating cardiac adaptation likely through impacts on the kinetics of targeting Rac1 for GEF-mediated activation at specific sarcolemmal domains in close proximity to its effectors.

*S*-palmitoylation cycling of Rac1 could potentially bifurcate cardiomyocyte Rac1 signaling into pathogenic signals controlling regulated reactive oxygen species (ROS) production by NOX2 that are associated with acceleration of cardiac pathology in response to stress (26, 56) and adaptive roles for *S*-acylated Rac1 in dampening sustained PKA signaling in response to β-adrenergic and other hypertrophic stress. Rho GDP-dissociation inhibitor (RhoGDI) is a cytosolic chaperone that regulates the delivery and extraction of all Rho family small GTPases, including Rac1, from their sites of action at cellular membranes by sequestering their C-terminal prenyl modification within its hydrophobic binding pocket (41, 43, 57, 58). Addition of a second C-terminal lipid modification to select Rho GTPases by *S*-palmitoylation can impair RhoGDI binding, resulting in a dually lipid-modified form that undergoes vesicular membrane transport rather than RhoGDI-dependent shuttling (36, 59–64), similar to H-Ras and N-Ras that are both *S*-palmitoylated and prenylated on their C-terminus (42, 64–66). Thus, *S*-palmitoylation could potentially target Rac1 to specific cardiomyocyte membrane domains to regulate unique effectors that are distinct from RhoGDI-chaperoned Rac1 or other Rac1 proteoforms that regulate its canonical pathogenic and pro-hypertrophic effectors, such as NOX2 (26, 56).

Elevated sympathetic nervous system (SNS) activity in cardiovascular disease enhances cardiac contractility primarily through enhanced cAMP-dependent PKA activity downstream of activation of cardiomyocyte β_1_ARs, but chronic stimulation of this pathway persistently increases cardiac workload and accelerates heart failure progression (16, 20, 67). Importantly, Rac1^cKI^ cardiomyocytes genetically resistant to Cys-178 *S*-palmitoylation not only exhibited markedly enhanced phosphorylation of PKA substrates in response to several chronic models of pathological hypertrophy but also had aberrant hyperphosphorylation of PKA substrates in response to acute β-adrenergic stimulation in vitro and in vivo. These findings collectively suggest that *S*-palmitoylation cycling of Rac1 has critical functions in sensing and responding to stress signals and restraining aberrant PKA-dependent remodeling of the E-C coupling phosphoproteome downstream of activated β-adrenergic receptors.

PKA-dependent phosphorylation in the heart is primarily reversed by the enzymatic activity of the protein phosphatases, PP1 and PP2A. PP2A functions as a heterotrimer with highly conserved “A” scaffolding and “C” catalytic subunits but a wide diversity of “B” regulatory subunits that confer substrate specificity (68). *Ppp2r3a* encodes 2 myocyte-enriched B family subunits, PR72 and PR130, each containing 2 EF-hand domains that impart calcium sensitivity and facilitate the ability of PP2A to counteract β-adrenergic–driven PKA activity via dephosphorylation of sarcomeric and calcium cycling protein substrates to restore myofilament relaxation and SR calcium cycling kinetics (22, 39, 40). Unbiased bulk RNA-seq performed early in the hypertrophic response to AngII identified unique and substantial downregulation of *Ppp2r3a* transcript levels in Rac1^cKI^ hearts that is further reduced following AngII infusion and PR72/PR130 protein levels are substantially reduced in Rac1^cKI^ hearts at baseline and in response to hypertrophic stress. Furthermore, PR72/PR130 localization, which is normally restricted to the M-line, is dysregulated in Rac1^cKI^ cardiomyocytes, providing potential mechanistic insight into the requirement of Rac1 *S*-palmitoylation to restrain hyperphosphorylation of the PKA-dependent phosphoproteome. *Ppp2r3a* mRNA and PR72 protein levels are also reduced in association with reduced systolic function in mice with cardiomyocyte-specific deletion of epidermal growth factor receptor (EGFR) and AAV9-mediated reintroduction of PR72 expression in cardiomyocytes normalized cardiac function (69), suggesting that downregulation of PR72 may be a targetable pathophysiologic mechanism common to cardiac maladaptation in response to multiple pathologic stressors. While we do not observe exacerbated total cellular PKA enzymatic activity in isoproterenol-treated Rac1^cKI^ myocytes, we cannot exclude the possibility that loss of Rac1 palmitoylation directly influences PKA activity and/or compartmentalization. Indeed, Rac1 has been shown to interact with the PKA regulatory subunit RII (70) and was thus proposed to act as an A-kinase anchoring protein (AKAP). While outside the scope of the current study, future investigations assessing the precise mechanisms by which loss of Rac1 *S*-palmitoylation elicits PKA substrate hyperphosphorylation through regulation of protein phosphatases and/or PKA are warranted.

**Figure 9:**
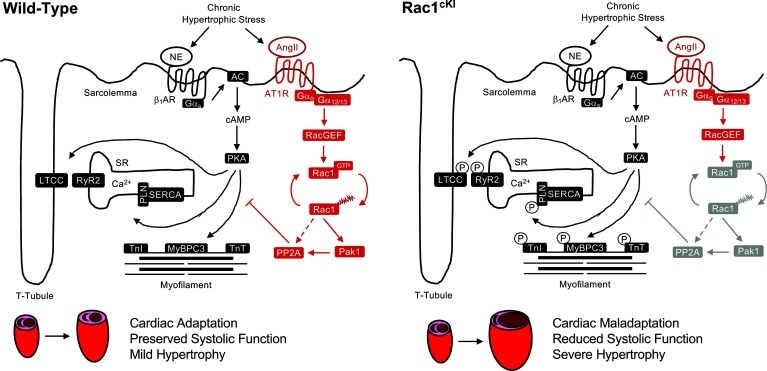
Working model of Rac1 Cys-178 palmitoylation in the regulation of cardiac remodeling and adrenergic signaling. Circulating levels of norepinephrine and angiotensin II are elevated during chronic hypertrophic stress. Activation of the β_1_AR elicits G_s_-dependent activation of adenylyl cyclase, leading to cAMP production and protein kinase A (PKA) activation. Active PKA phosphorylates substrates critical for excitation-contraction coupling. AT1R activation leads to Rac1 activation and promotes Rac1 *S*-palmitoylation cycling. *S*-palmitoylated Rac1 is required to evoke dephosphorylation of PKA substrates and dampen excessive adrenergic signaling that promotes cardiac maladaptation, likely through Pak1-dependent activation of PP2A. Upon loss of Rac1 palmitoylation cycling at Cys-178 (as observed in Rac1^cKI^ hearts), PP2A-mediated antagonism of PKA substrate activity is not properly regulated, resulting in hyperphosphorylation of PKA substrates that chronically promotes cardiac decompensation, systolic dysfunction, and adverse hypertrophic remodeling. AC, adenylyl cyclase; AngII, angiotensin II; AT1R, angiotensin II receptor type I; β_1_AR, β1 adrenergic receptor; LTCC, L-type calcium channel; MyBPC3, myosin binding protein C3; NE, norepinephrine; Pak1, p21-activated kinase-1; PKA, protein kinase A; PLN, phospholamban; PP2A, protein phosphatase 2A; SERCA, sarco-endoplasmic reticulum calcium ATPase; TnI, troponin I; TnT, troponin T.

Taken together, data presented here indicate that *S*-palmitoylation of Rac1 at Cys-178 is necessary to counteract aberrant PKA activity in response to hypertrophic stress and suggest *S*-palmitoylated Rac1 could potentially target PR72/PR130 to distinct cardiomyocyte compartments, directly or indirectly, for regulated dephosphorylation of myofilament and calcium cycling protein substrates of PKA to restore physiologic E-C coupling in the face of persistent adrenergic stimulation. Pak proteins are canonical effectors of Rac1 and cardiomyocyte-specific deletion of either Pak1 or Pak2 phenocopies exacerbated cardiac hypertrophy and dysfunction in response to pressure overload or AngII infusion (71, 72) that we observed in Rac1^cKI^ mice. Both Pak1 and Pak2 are reported to interact with and activate PP2A in cardiomyocytes (39, 72) and modulate calcium handling and contractility (73, 74). Thus, *S*-palmitoylation of Rac1 could deploy Pak1 or Pak2 in cardiomyocytes to indirectly facilitate dephosphorylation of PKA targets by PP2A in response to cardiac stressors rather than direct interaction of *S*-palmitoylated Rac1 with PR72 or PKA. Regardless of the precise mechanism, we propose that a Rac1 *S*-palmitoylation cycle directly or indirectly represses sustained pathogenic PKA signaling in response to cardiovascular stress signals such as sustained activation of the RAS and SNS (Figure 9).

Loss of PKA in cardiomyocytes has no deleterious effects on cardiac structure or function but mitigates tachycardia and inotropic responses to β-adrenergic stimulation (16) and genetic inhibition of PKA prevents pressure overload–induced heart failure (20). Conversely, constitutive activation of PKA in cardiomyocytes causes a severe heart failure phenotype (67), suggesting alternative strategies to repress or antagonize PKA activity, such as enhancement of Rac1 *S*-palmitoylation or PR72-regulated phosphatase activity, could provide a more targeted means to repress pathogenic PKA activity and diminish cardiac hypertrophy and decompensation in response to sustained β-adrenergic signaling (Figure 9) without the untoward side effects of beta blockers or substantial cAMP-dependent effects on heart rate and conduction velocity.

## Methods

### Sex as a biological variable.

In all mouse experiments, we used approximately equal numbers of both male and female mice. The specific number of male and female mice in each group for phenotyping experiments can be found in the supplemental tables. For NRCM experiments, hearts from both male and female rat pups were pooled at the time of collection and plated as a mixed population of cells, with each preparation representing 1 biological replicate. Whenever possible, we examined sex-specific effects; however, no statistically significant differences between sexes were found in any of the data included within this paper.

### Molecular cloning and viral production.

Detailed descriptions of plasmid and virus generation are available in the Supplemental Methods.

### Animals and pathologic hypertrophy models.

Rac1^cKI^ mice were generated on a pure C57BL/6J genetic background by Ozgene as follows. Gene targeting of the *Rac1* locus was performed in ES cells using a minigene approach to replace endogenous exons 5 through 6 with a *loxP*-targeted fused WT exon 5/6 followed by WT exon 5 and exon 6 encoding the C178S mutation to evoke expression of the Rac1^C178S^ mutant protein from the endogenous locus upon Cre-mediated recombination, as illustrated in Figure 2A. Germline mice with the conditional Rac1-knockin allele (Rac1^cKI^) were obtained and crossed to a ubiquitous Cre line (OzCre; PGK-Cre in *ROSA26* locus, *Gt(ROSA)26Sor^tm1(PGK1-cre)^*Ozg) on a C57BL/6J genetic background to generate whole-body Rac1-knockin mice. Rac1^cKI^ mice were crossed to the transgenic αMHC promoter–driven Cre line (75) (*Myh6*-Cre, The Jackson Laboratory, strain 011038, B6N.FVB(B6)-Tg(Myh6-cre)2182Mds/J) on a C57BL/6J genetic background to knock in the Rac1 C178S mutation specifically in cardiomyocytes. Rac1^cKI^ mice were crossed with cardiomyocyte-specific Tg mice overexpressing the human AT1R under control of the *Myh6* promoter (10) (gift from Mona Nemer, University of Ottawa, Ottawa, Ontario, Canada). TgAT1R mice were initially generated on a mixed C57BL/6J × C3H genetic background decades ago (10) and experimental animals from the TgAT1R cross with Rac1^cKI^ mice were on an identical mixed C57BL/6J × C3H genetic background that is predominantly C57BL/6J.

Osmotic pumps (Alzet, 1007D or 1002) containing saline or AngII (3 mg/kg/day; Sigma-Aldrich, A9525) were subcutaneously implanted for 3 or 14 days of infusion. Sham surgeries or TAC surgeries to induce left ventricular pressure overload in 8- to 12-week-old mice were performed at the UM Physiology Phenotyping Core, as previously described (76–78). Postconstriction aortic velocities were measured by pulse-wave Doppler 8 weeks after surgery and confirmed to be within the same range for all genotypes (Supplemental Table 3). For acute treatment, isoproterenol (Sigma-Aldrich, I6504) was diluted in sterile saline and injected intraperitoneally at a dose of 300 mg/kg. For overexpression studies, 1 × 10^12^ viral genomes of AAV9 were injected into the mediastinum of WT CD1 mice (Charles River Laboratories), or C57BL/6J mice (The Jackson Laboratory) in the case of proximity labeling proteomic studies, on postnatal day 6.

### Echocardiography.

Murine cardiac structure and function were evaluated by echocardiography using a Visual Sonics Vevo F2LT ultrasound machine with a 46 MHz transducer (Fujifilm) as described previously (46, 78–80). M-Mode images were taken in the parasternal short axis at the level of the papillary muscle. Mice were anesthetized with 3% isoflurane and maintained at 1%–2% isoflurane throughout the imaging process. Measurements and analyses were conducted using VevoLab software (Fujifilm).

### Histology.

Hearts were fixed overnight at 4ºC in 4% paraformaldehyde in phosphate-buffered saline (PBS). After fixation, hearts were dehydrated in ethanol and embedded in paraffin, sectioned, and stained with hematoxylin & eosin (H&E) or Picrosirius red (PSR) at the University of Michigan Dental School Histology Core. Percentage interstitial fibrosis was quantified using ImageJ (NIH) as described previously (77, 81, 82). Briefly, four ×10-magnified images of PSR-stained left ventricular free wall per animal were analyzed and the mean value was calculated as an index of fibrosis.

### Primary cardiomyocyte isolation and culture.

AMCMs were isolated from 2- to 4-month-old mice using the Langendorff-free method, as previously described (83). Following perfusion with a collagenase buffer and calcium reintroduction, AMCMs were plated on laminin-coated (Sigma-Aldrich, L2020) dishes, chamber slides, or coverslips in plating media consisting of phenol red–free Medium 199 (M199; Gibco, 11043-023) with 5% fetal bovine serum (FBS; Sigma-Aldrich, F4135), 10 mM 2,3-butanedione monoxime (BDM; Sigma-Aldrich, B0753), and 1% penicillin-streptomycin (P/S; Hyclone, SV30010). After 2–3 hours, plating media containing unattached cells were removed and replaced with AMCM culture media consisting of M199, 0.1% bovine serum albumin (BSA; Sigma-Aldrich, A9647), 10 mM BDM, 1× insulin-transferrin-selenium (ITS; Invitrogen, 41400045), 1× chemically defined lipid concentrate (Gibco, 11905-031), and 1% P/S. For immunoblotting experiments, AMCMs were serum-starved overnight before acute treatment with 10 μM isoproterenol (Sigma-Aldrich, I6504) diluted in AMCM culture media for 5 minutes before harvesting cells.

NRCMs were isolated from Sprague-Dawley rat pups on postnatal days 1–2 using the Neonatal Cardiomyocyte Isolation System (Worthington, LK003300), following the established protocol (46). NRCMs were transduced with adenoviruses in serum-free DMEM (Corning, 10-013-CV) for 4–6 hours and then maintained in M199 (Corning, 10-060-CV) supplemented with 1% bovine growth serum (BGS; Cytiva, SH30541) and 1% P/S. NRCMs were harvested 48 hours after transduction or at 24 hours after transduction were changed to fresh serum-free M199 with or without AngII (10 μM) for an additional 48 hours before harvesting for Rac1 activity assays as described below.

### Preparation of cell and tissue lysates and immunoblotting.

For Western blotting, cell or tissue lysates were prepared in radioimmunoprecipitation assay (RIPA) buffer (50 mM Tris HCl pH 7.4, 1% Triton X-100, 1% sodium deoxycholate, 1 mM EDTA, and 0.1% SDS) supplemented with protease inhibitors (100 mM AEBSF, 1 mg/mL leupeptin, 1 mg/mL aprotinin) and phosphatase inhibitors (2 mM sodium fluoride and 1 mM sodium orthovanadate). Mouse hearts were homogenized in a bead mill homogenizer (Next Advance) and homogenates sonicated and clarified by centrifugation at 13,523*g* at 4ºC for 10 minutes. Samples were boiled at 95ºC for 5 minutes in Laemmli buffer, separated by SDS-PAGE (BioRad), and then transferred to polyvinylidene difluoride (PVDF) membranes (Millipore, Immobilon-FL, IPVH00010). Primary antibodies were applied and incubated overnight at 4ºC, as previously described (44). The PVDF membranes were incubated with IRDye-conjugated secondary antibodies (LI-COR; 1:5,000–1:15,000) and imaged using an Odyssey CLx imaging system (LI-COR). The primary antibodies used for Western blotting are listed in the supplemental material. To assess total protein loading following immunoblotting, PVDF membranes were stained with amido black staining solution (4% v/v ethanol, 1% v/v acetic acid, 0.1% w/v amido black 10B [Thermo Fisher Scientific, BP124]) for 2 minutes and destained in a solution containing 40% v/v ethanol, 10% v/v acetic acid, and 2% v/v glycerol before imaging. Amido black intensity was quantified in ImageJ (NIH). Alternatively, PVDF membranes were stained with Ponceau solution (5% v/v acetic acid, 0.1% w/v Ponceau S [Thermo Fisher Scientific, 161470100]) or acrylamide gels were stained with LC6060 SimplyBlue SafeStain (Invitrogen, 465034).

### Rac1 activity assays.

To assess steady-state Rac1 activity in vivo, hearts were homogenized in activity assay buffer (25 mM HEPES pH 7.5, 150 mM NaCl, 1% NP-40, 10 mM MgCl_2_, 1 mM EDTA, and 2% glycerol) supplemented with protease and phosphatase inhibitors as above and clarified by centrifugation. After centrifugation, equal amounts of protein were removed for input loading. Equal amounts of remaining protein were incubated with magnetic beads coupled to the p21-binding domain of p21-activated kinase (PAK-PBD) (Millipore, 17-283), which specifically binds to GTP-bound Rac1 and Cdc42, for 45 minutes at 4ºC. The beads were washed 3 times in activity assay buffer and heated at 95ºC for 5 minutes to elute proteins. Both eluates and input samples were subjected to SDS-PAGE and probed with a Rac1-specific or FLAG epitope–specific primary antibody.

For experiments in NRCMs with overexpressed Rac1, we generated a recombinant glutathione *S*-transferase (GST)-PAK-PBD fusion protein by transforming BL21-DE3 *E*. *coli* with a pGEXT-PAK-PBD-70-117 plasmid that contains residues 70–117 of human Pak1 (Addgene, 12217, gift from Jonathan Chernoff). The BL21-DE3 cultures were incubated with 1 mM isopropyl-β-D-thiogalactopyranoside (IPTG; Thermo Scientific, R0392) to stimulate protein production and the GST-PAK-PBD protein was extracted in lysis buffer (10 mM Tris pH 8.0, 1 mM EDTA, 150 mM NaCl, 10 mM DTT, and 1.2% Sarkosyl supplemented with protease and phosphatase inhibitors), sonicated, and centrifuged. The supernatant was then supplemented with Triton X-100 to a final concentration of 2.5% Triton X-100 and rocked for 30 minutes at room temperature at which point lysates were then aliquoted and stored at –80ºC. PAK-PBD protein–containing lysates were coupled to glutathione–Sepharose 4B beads (GE Healthcare, 17-0756-05) for GST pull-down assays as described previously (44, 46). Clarified NRCM lysates were incubated with GST-PAK-PBD–coupled glutathione-Sepharose beads for 45 minutes at 4ºC and processed as described above.

### S-palmitoylation pull-down assays.

Acyl-biotin exchange was performed to assess steady-state levels of *S*-palmitoylation. Cell or tissue lysates were prepared in RIPA buffer as above and mixed with an equal volume of 2× free thiol–blocking solution (100 mM HEPES, 1 mM EDTA, 5% SDS, 0.6% methyl methanethiosulfonate [MMTS]) for 30 minutes at 37ºC. The proteins were precipitated with acetone overnight and resulting pellets were washed with 70% acetone 3 times and resuspended in buffer containing 100 mM HEPES, 1 mM EDTA, and 1% SDS. Once resolubilized, the lysates were incubated with a final concentration of 500 mM hydroxylamine (NH_2_OH) and 0.4 mM biotin-HPDP at room temperature for 1 hour, followed by acetone precipitation overnight. As a negative control, one sample was mixed with 500 mM Tris pH 7.4 instead of NH_2_OH. Protein pellets were washed 3 times with 70% acetone and then subjected to pull-down with high-capacity streptavidin-agarose beads (Pierce, 20359) overnight at 4ºC in pull-down buffer (100 mM HEPES, 1 mM EDTA, 0.5% SDS, and 0.2% Triton X-100). Subsequently, beads were washed 3 times in pull-down buffer, boiled for 5 minutes in Laemmli buffer, and analyzed by SDS-PAGE and Western blotting as described previously (44, 46). Caveolin-1 was used as a positive control for *S*-palmitoylation.

Acyl-RAC was alternatively used to affinity purify *S*-palmitoylated proteins, as described previously (76). Briefly, RIPA lysates were treated with MMTS to block free thiols and protein precipitated as described above. Protein pellets were resolubilized in 100 mM HEPES (pH 7.4), 1 mM EDTA, and 1% SDS and incubated with a final concentration of 150 mM Tris (pH 7.4) (negative control) or 150 mM NH_2_OH in the presence of thiopropyl–Sepharose 6B beads (Sigma-Aldrich, T8387). Beads were washed 4 times with 100 mM HEPES pH 7.4, 1 mM EDTA, and 0.3% SDS and *S*-palmitoylated proteins eluted by boiling in Laemmli buffer and analyzed by SDS-PAGE.

### RNA-seq and qPCR.

Whole heart RNA was isolated using the RNeasy Mini Kit (Qiagen) according to the manufacturer’s instructions. Bulk RNA-seq and pathway analyses were performed by Novogene. Sequencing was performed on an Illumina NovaSeq PE150 platform and data mapped to the GRCm39/mm39 reference genome. FeatureCounts (84) v1.5.0-p3 was used to count the numbers of reads mapped to each gene and fragments per kilobase of transcript sequence per millions base pairs sequenced (FPKM) were calculated for each gene. Differential expression analyses were performed using the DESeq2R package (1.20.0) (85) and Gene Ontology enrichment analyses of differentially expressed genes was conducted using by the clusterProfiler R package.

For qPCR, RNA was converted to cDNA with the High-Capacity cDNA Reverse Transcription Kit (Thermo Fisher Scientific, 4368814). The qPCR reactions were performed with primers listed in Supplemental Table 7 and Power Up SYBR Green (Applied Biosystems, A25742) on a QuantStudio 7 Flex Real-Time PCR System (Applied Biosystems), as described previously (46, 76).

### Statistics.

All statistical analyses were performed in GraphPad Prism and the relevant statistical test(s) performed on each data set are detailed in the figure legends. Statistical significance was considered when *P* values were less than 0.05. All data in bar graphs are plotted as the mean ± SEM.

### Study approval.

The animal studies in this manuscript were approved and overseen by the University of Michigan Institutional Animal Care and Use Committee (IACUC) and adhered to the NIH *Guide for the Care and Use of Laboratory Animals* (National Academies Press, 2011).

### Data availability.

Values for all data points in graphs are reported in the Supporting Data Values file. RNA-seq data have been deposited in the NCBI Gene Expression Omnibus (GEO) database and are accessible through GEO Series accession number GSE305462. Proteomics data have been deposited to the MassIVE database (accession number MSV000098836) and are publicly available on ProteomeXchange (dataset identifier PXD067386).

## Author contributions

JPT, RES, AS, and MJB performed experiments and collected data. MJB and NM provided reagents. JPT, RES, AS and MJB analyzed data. JPT and MJB wrote the manuscript. All authors edited and proofread the manuscript before submission.

## Funding support

This work is the result of NIH funding, in whole or in part, and is subject to the NIH Public Access Policy. Through acceptance of this federal funding, the NIH has been given a right to make the work publicly available in PubMed Central.

National Heart, Lung, and Blood Institute, grant R01HL167778, to MJB.

National Heart, Lung, and Blood Institute, grant F31HL165180, to JPT.

American Heart Association, grant 898429, to JPT.

## Supplementary Material

Supplemental data

Unedited blot and gel images

Supporting data values

## Version 1 09/09/2025

In-Press Preview

## Version 2 10/22/2025

Electronic publication
