# Supplementary material for: Rac1 palmitoylation is required for cardiac stress adaptation and regulation of protein kinase A signaling
Source: JCI Insight. 2025 Sep 9;10(20):e193733. doi: 10.1172/jci.insight.193733 (PMC12581661; doi:10.1172/jci.insight.193733)
Supplement: Supplemental data [file jciinsight-10-193733-s055.pdf]

SUPPLEMENTAL MATERIAL

Rac1 palmitoylation is required for cardiac stress adaptation and regulation of protein kinase A signaling

James P. Teuber[1], Rachel E. Scissors[1], Arasakumar Subramani[1], Nageswara Madamanchi[2], and Matthew J. Brody[1,2]

[1] Department of Pharmacology, University of Michigan, Ann Arbor, MI, USA

[2] Division of Cardiovascular Medicine, Department of Internal Medicine, University of Michigan, Ann Arbor, MI, USA

Corresponding Author:

Matthew J. Brody, Ph.D.

Department of Pharmacology

University of Michigan Medical School

1150 W. Medical Center Drive

Ann Arbor, MI 48109-5632

Phone: (734) 763-3255

Email: majbrody@umich.edu

Supplemental Methods

Molecular Cloning and Viral Production. Mouse Rac1 cDNA (Horizon Discovery, Cat #MMM1013-202762775) was cloned into the p3xFLAG-CMV-10 vector (Sigma-Aldrich, Cat #E7658). The sequence encoding N-terminally Flag-tagged Rac1 was then subcloned into either the pacAd5-CMV-K-pN adenoviral shuttle vector (Cell Biolabs, Cat #325201) or the pAV-cTnT (Vigene Biosciences) for production of cardiomyocyte-specific AAV9, as previously described (1). The Cys178Ser mutation was introduced into the Rac1 coding sequence using the Quick Change II XL Site-Directed Mutagenesis kit (Agilent, Cat #200521) according to the manufacturer's instructions. Wildtype Rac1 and Rac1^{C178S} mutant cDNAs were also cloned into the myc-BioID2-MCS vector (Addgene plasmid #74223, kind gift from Dr. Kyle Roux) (2) and the resultant cDNAs encoding Rac1 fusion proteins with an N-terminal BioID2 for biotinylation-mediated proximity labeling as well as a BioID2-tagged Venus-CAAX control (3) were similarly subcloned into the pAV-cTnT vector (Vigene Biosciences) for generation of cardiomyocyte-specific AAV9s (1). The accuracy of all constructs was verified by DNA sequencing. Recombinant adenovirus was produced with the RAPd CMV Adenoviral Expression System (Cell Biolabs, Cat #VPK-252) as described (4, 5) and AAV9s were manufactured by Vigene Biosciences. The β-galactosidase (β-Gal) control adenovirus (4, 6) and the cardiomyocyte-specific luciferase control AAV9 (1) have been previously reported.

PKA activity assay. To assess PKA enzymatic activity, we used the commercially available plate-based PKA activity assay kit (Arbor Assays, Cat #K027-H1). Briefly, AMCMs were isolated as described above and treated with saline or isoproterenol for 5 minutes in culture media. AMCMs were lysed and diluted in kinase assay buffer and, along with ATP, added to a plate coated with immobilized PKA substrate. Following incubation, phosphorylation of the immobilized PKA substrate was detected by an anti-phospho-PKA substrate antibody and subsequent incubations with HRP-conjugated secondary antibody and TMB substrate followed by measurement of OD450 with a SpectraMax Plus plate reader (Molecular Devices). OD450 measurements were fit to a PKA standard curve to quantify PKA activity.

Subcellular cytosol and membrane fractionation. Cytosolic- and membrane-enriched fractions were prepared as described previously (7). Ventricular tissue was solubilized in lysis buffer (50 mM Tris pH 7.5, 5 mM EGTA, 2 mM EDTA, 5 mM DTT, 0.05% digitonin supplemented with protease and phosphatase inhibitors as above) in a bead mill homogenizer (Next Advance) and homogenates cleared by centrifugation at 14,000 x *g* for

15 minutes at 4ºC. Supernatants were collected as cytosol-enriched fractions and insoluble pellets were resuspended in lysis buffer with 1% Triton X-100 for 10 minutes on ice before clearing by centrifugation as above and collecting supernatants as membrane-enriched fractions.

Sucrose density gradient lipid raft fractionation. Hearts were ground in a mortar and pestle on dry ice and Dounce homogenized in sodium bicarbonate/EDTA buffer (250 mM Na_2CO_3 pH 11.0 with 2 mM EDTA). Homogenates were sonicated for three 20-second pulses on ice and nuclei cleared by centrifugation at 1,000 x *g* for 10 minutes at 4ºC. The post-nuclear supernatant (2 mL) was mixed with an equal volume of 80% sucrose in 2-(N-morpholino)ethanesulfonic acid (MES) buffer (25 mM MES, 150 mM NaCl, 2 mM EDTA, 80% sucrose) and loaded into the bottom of a 13 mL ultra-clear centrifuge tube (Beckman Coulter, Cat #344059). Subsequent layers (4.5 mL each) of 30% sucrose in MES buffer (12.5 mM MES, 75 mM NaCl, 2 mM EDTA, 150 mM Na_2CO_3, 30% sucrose) and 5% sucrose in MES buffer (12.5 mM MES, 75 mM NaCl, 2 mM EDTA, 150 mM Na_2CO_3, 5% sucrose) were loaded above the sample. The tubes were spun in a SW41 rotor for 20-24 hours at 41,000 rpm at 4ºC. Following centrifugation, a cloudy interface between fractions 4 and 5 appeared indicating the presence of caveolin-enriched lipid rafts. Twelve (1 mL) fractions were taken from the top to bottom (low to high sucrose concentrations), mixed with Laemmli buffer, and analyzed by SDS-PAGE. For the relative quantification of endogenous Rac1, fraction 5 (rafts) and fractions 10-12 (cytosolic, non-rafts) were subjected to chloroform-methanol precipitation and SDS-PAGE. The levels of Rac1 in rafts were normalized to caveolin-1 while the levels of Rac1 in the non-rafts were normalized to GAPDH.

Proximity labeling proteomics. Proximity labeling proteomics were performed using the BioID2 fusion protein system (2). Mice were injected with AAV9s encoding troponin T promoter-driven BioID2 fusion proteins (myc-BioID2-Venus-CAAX as control, myc-BioID2-Rac1-WT, and myc-BioID2-Rac1-C178S) at postnatal day 6. At 2 months of age, mice were fed *ad libitum* with high biotin chow (Teklad, Cat #TD.02458). Forty-eight hours post diet change, mice were sacrificed and hearts excised, rinsed in ice-cold PBS, and flash frozen in liquid nitrogen. Left ventricular tissue was homogenized in modified RIPA buffer (50 mM Tris, 150 mM NaCl, 0.1% SDS, 0.5% sodium deoxycholate, 1% Triton X-100, pH 7.5) in a beadmill homogenizer (Next Advance) and sonicated. Lysates were cleared by centrifugation at 12,000 rpm for 10 minutes at 4ºC. Lysates (4.5 mg) were incubated with streptavidin-coated magnetic beads (Fisher Scientific, Cat #88817) with end-over-end rotation at 4ºC for 18-20 hours then washed twice in modified RIPA buffer, once in 1 M KCl, 0.1 M Na_2CO_3, 2% SDS in Tris

pH 7.5, and then in 2 M urea in 10 mM Tris pH 8.0 followed by two more washes in modified RIPA buffer. Lastly, beads were washed in ice-cold 1X PBS and flash frozen in liquid nitrogen prior to proteomics analysis. On-bead digestion and subsequent liquid chromatography with tandem mass spectrometry (LC-MS/MS) analyses were performed exactly as described previously at the University of Michigan Proteomics Resource Facility (3). Briefly, samples were reduced, alkylated, trypsin digested, and reconstituted samples were labeled with TMT 16-plex reagents (Thermo Scientific, Cat #A44521) and subjected to LC-MS/MS. Sequenced peptides were mapped to the mouse proteome and abundance normalized to total sequenced peptides.

Assessment of myocardial superoxide production. Hearts were embedded in OCT compound and flash frozen without fixation in bubbling 2-methylbutane over dry ice. Cryosections (8 μm) were prepared and stored at -80ºC overnight. The next day, sections were thawed, washed with PBS, stained with 50 μM dihydroethidium (DHE, Invitrogen, Cat #) for 30 minutes at 37ºC, mounted with ProLong Gold mounting media (Invitrogen, Cat #P36930) and imaged immediately on a Nikon Zeiss LSM 880 scope. Nine images in separate regions of the left ventricular myocardium were taken for each mouse and average mean intensity calculated as an index of oxidative stress.

Immunostaining. For immunostaining, AMCMs plated on laminin-coated chamber slides (Ibidi, Cat #80826) or coverslips were fixed in 4% PFA, incubated in blocking buffer (5% goat serum, 1% BSA, 1% glycine, 0.2% Triton X-100 in PBS pH 7.4) for one hour, and immunostained with anti-Rac1 (Novus, Cat #NB100-91266, 1:75), anti-PR72/PR130 (Atlas Antibodies, Cat #HPA035829, 1:40), and/or anti-α-actinin (Sigma, Cat #A7811, 1:100) primary antibodies diluted in blocking buffer overnight at 4ºC. Cells were washed with PBS containing 0.1% NP-40 and incubated with AlexaFluor-conjugated secondary antibodies (Thermo Fisher) diluted in blocking buffer for 1-2 hours at room temperature. Immunofluorescent imaging performed using a Nikon Zeiss LSM 880 confocal microscope. Plot profiles presented in Supplemental Figure 8 were generated in ImageJ (NIH).

Antibodies. The primary antibodies used for Western blotting were as follows: Rac1 (BD Biosciences, Cat #610650, clone 102, 1:500), GAPDH (Fitzgerald/Biosynth, Cat #10R-G109A, clone 6C4, 1:20,000-1:50,000), Flag (Sigma-Aldrich, Cat #F1804, clone M2, 1:1000), caveolin-1 (Cell Signaling, Cat #3267, clone D46G3, 1:20,000), phospho-PKA substrates (RRXS*/T* motif, Cell Signaling, Cat #9624, clone 100G7E, 1:1000), and PR72/PR130 (Atlas Antibodies, Cat #HPA035829, 1:1000).

References for Supplemental Material

1. Grimes KM, Prasad V, Huo J, Kuwabara Y, Vanhoutte D, Baldwin TA, et al. Rpl3l gene deletion in mice reduces heart weight over time. *Front Physiol.* 2023;14:1054169.
2. Kim DI, Jensen SC, Noble KA, Kc B, Roux KH, Motamedchaboki K, et al. An improved smaller biotin ligase for BioID proximity labeling. *Mol Biol Cell.* 2016;27(8):1188-96.
3. Chandan NR, Abraham S, SenGupta S, Parent CA, and Smrcka AV. A network of Galpha(i) signaling partners is revealed by proximity labeling proteomics analysis and includes PDZ-RhoGEF. *Sci Signal.* 2022;15(717):eabi9869.
4. Brody MJ, Schips TG, Vanhoutte D, Kanisicak O, Karch J, Maliken BD, et al. Dissection of Thrombospondin-4 Domains Involved in Intracellular Adaptive Endoplasmic Reticulum Stress-Responsive Signaling. *Mol Cell Biol.* 2016;36(1):2-12.
5. Essandoh K, Subramani A, Ferro OA, Teuber JP, Koripella S, and Brody MJ. zDHHC9 Regulates Cardiomyocyte Rab3a Activity and Atrial Natriuretic Peptide Secretion Through Palmitoylation of Rab3gap1. *JACC Basic Transl Sci.* 2023;8(5):518-42.
6. Essandoh K, Eramo GA, Subramani A, and Brody MJ. Rab3gap1 palmitoylation cycling modulates cardiomyocyte exocytosis and atrial natriuretic peptide release. *Biophys J.* 2025.
7. Liu R, Correll RN, Davis J, Vagnozzi RJ, York AJ, Sargent MA, et al. Cardiac-specific deletion of protein phosphatase 1beta promotes increased myofilament protein phosphorylation and contractile alterations. *J Mol Cell Cardiol.* 2015;87:204-13.

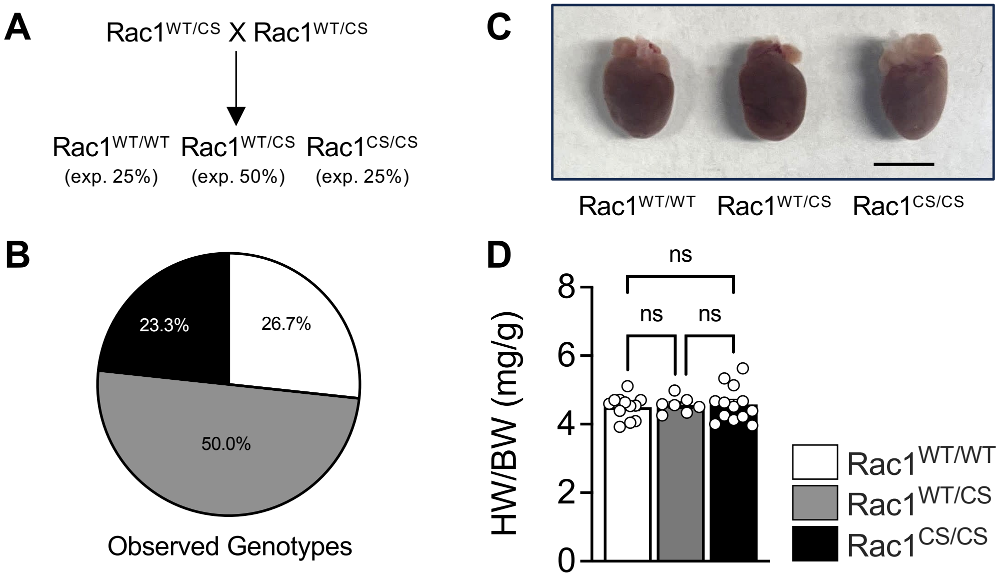

Supplemental Figure 1. Global loss of Rac1 S-palmitoylation at cysteine-178 is dispensable for proper development. (A) Breeding schematic. Heterozygous $Rac1^{WT/CS}$ mice were crossed to generate $Rac1^{WT/WT}$, $Rac1^{WT/CS}$, and $Rac1^{CS/CS}$ mice. **(B)** Percentage of observed offspring of the indicated genotypes from heterozygous crosses**.** n=322 mice. (**C**) Representative whole heart images and **(D)** heart weight-to-body weight (HW/BW) ratios of the indicated genotypes of mice at 4 months of age. n=7-13 mice/group. NS=not significant, one-way ANOVA,

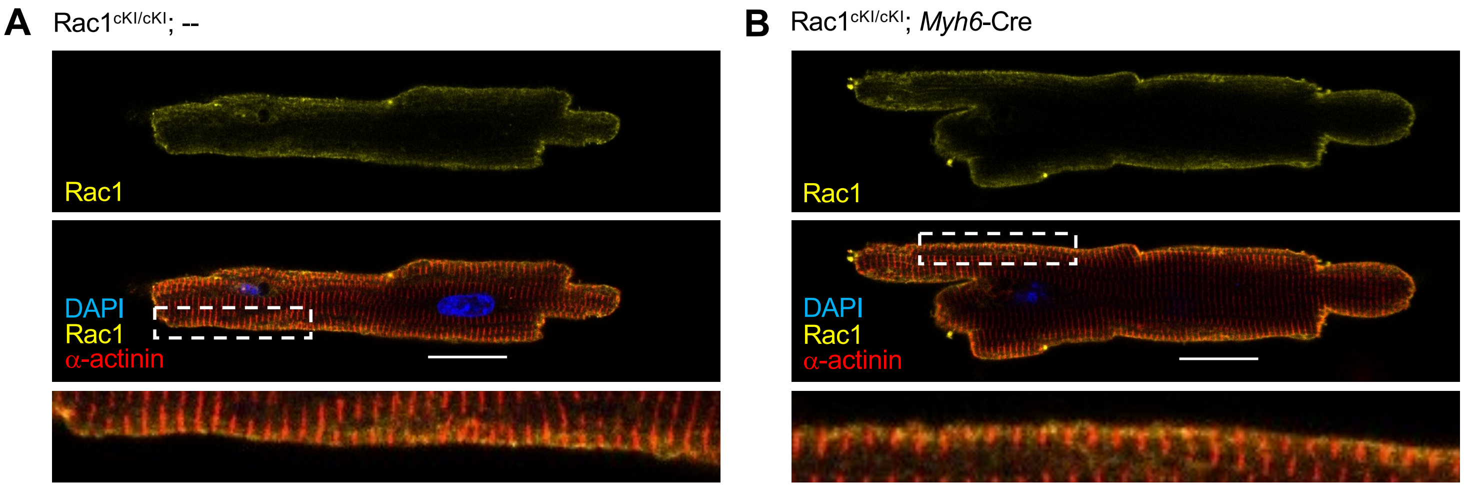

Supplemental Figure 2. Rac1 localization in control and Rac1^{cKI} cardiomyocytes. Representative immunocytochemistry of Rac1 and α-actinin in **(A)** control or **(B)** Rac1^{cKI} cardiomyocytes showing striated Rac1 staining along the sarcolemma in myocytes isolated from mice of both genotypes. Rac1 is pseudocolored yellow, alpha-actinin pseudocolored red, and DAPI was used to stain nuclei blue. Enlargement of region in the sarcolemma in the dashed box in the middle panel is shown in the inset at the bottom. Scale bar = 20 μm.

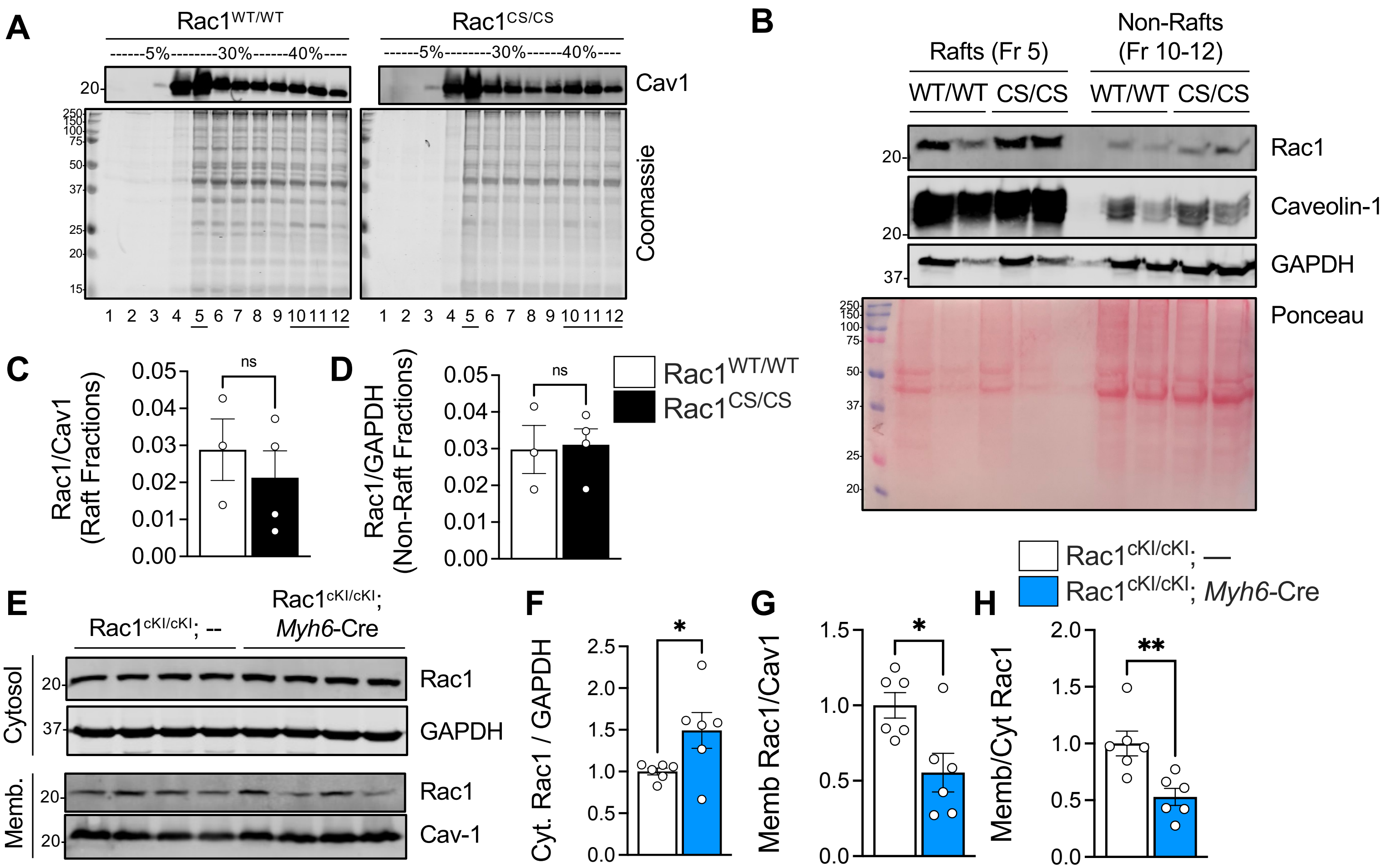

Supplemental Figure 3. Palmitoylation-deficient Rac1^{C178S} is not differentially partitioned to lipid rafts but is less associated with cellular membranes in Rac1^{cKI} hearts. (A) Purification of caveolin-rich lipid rafts by sucrose gradient fractionation. Twelve (1 mL) fractions were obtained from low (5%) to high (40%) sucrose in Rac1$^{WT/WT}$ and Rac1$^{CS/CS}$ hearts. Caveolin-rich lipid rafts were obtained in fractions 4-5. **(B)** Proteins were precipitated by chloroform-methanol precipitation from fraction 5 containing lipid rafts and fractions 10-12 containing non-rafts in hearts of each genotype. Following precipitation and resuspension, fractions were subjected to immunoblotting for Rac1, caveolin-1, and GAPDH with Ponceau stain used to assess total protein loading. **(C)** Rac1 raft levels normalized to caveolin-1 (Cav1) and **(D)** Rac1 non-raft levels normalized to GAPDH, NS=not significant by unpaired t-test. **(E)** Representative immunoblots of Rac1 in cytosolic-enriched and membrane-enriched fractions from 2-3-month-old control or Rac1^{cKI} mouse ventricular tissue. GAPDH was used as a cytosolic loading control and caveolin-1 as a membrane loading control. Quantification of **(F)** cytosolic Rac1 normalized to GAPDH, **(G)** membrane Rac1 normalized to caveolin-1, and **(H)** membrane-to-cytosolic Rac1 ratio. *p<0.05, **p<0.01, unpaired t-test.

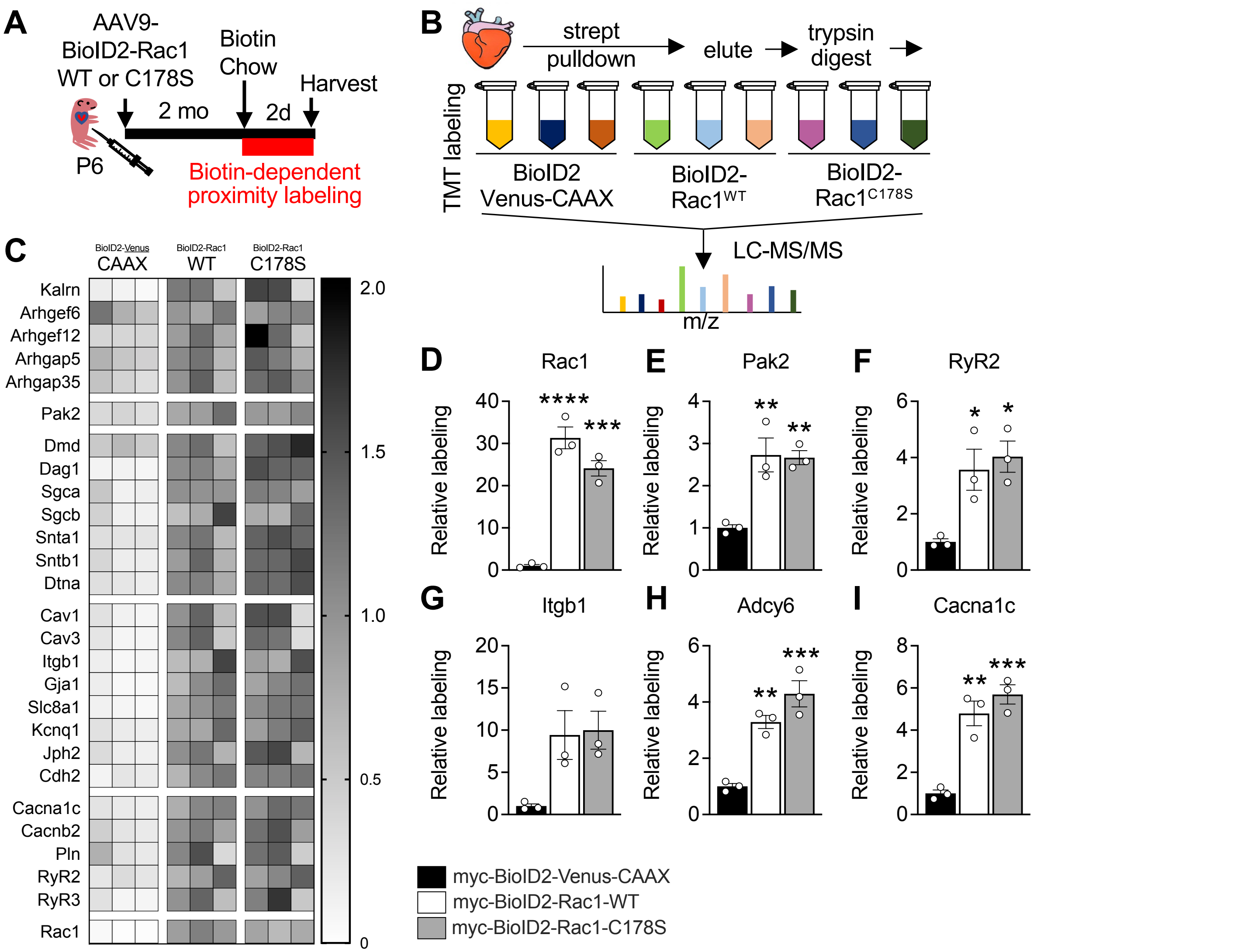

Supplemental Figure 4. Proximity-labeling proteomics of wildtype Rac1 and palmitoylation-deficient Rac1^{C178S} in cardiomyocytes in vivo. (A, B) Experimental schematic. Mice were injected at postnatal day 6 (P6) with cardiomyocyte-specific AAV9s encoding BioID2-Venus-CAAX, BioID2-Rac1-WT, or BioID2-Rac1-C178S. At 2 months of age, mice were fed high biotin chow for 2 days and hearts were collected and biotinylated proteins purified by pulldown with streptavidin-coated beads. Eluted proteins were subjected to trypsin digest, TMT labeling, and LC-MS/MS. **(C)** Heat map of proteins with enriched labeling in hearts of mice expressing BioID2-tagged Rac1 constructs over Venus-CAAX control normalized to average labeling in hearts expressing BioID2-Rac1-WT. Quantification of proximity labeling of **(D)** Rac1, **(E)** Pak2, **(F)** RyR2, **(G)** Itgb1, **(H)** Adcy6 and **(I)** Cacna1c in hearts of the indicated mice. *$p<0.05$, **$p<0.01$, ***$p<0.001$, ****$p<0.0001$, one-way ANOVA with post-hoc Tukey's multiple comparisons test.

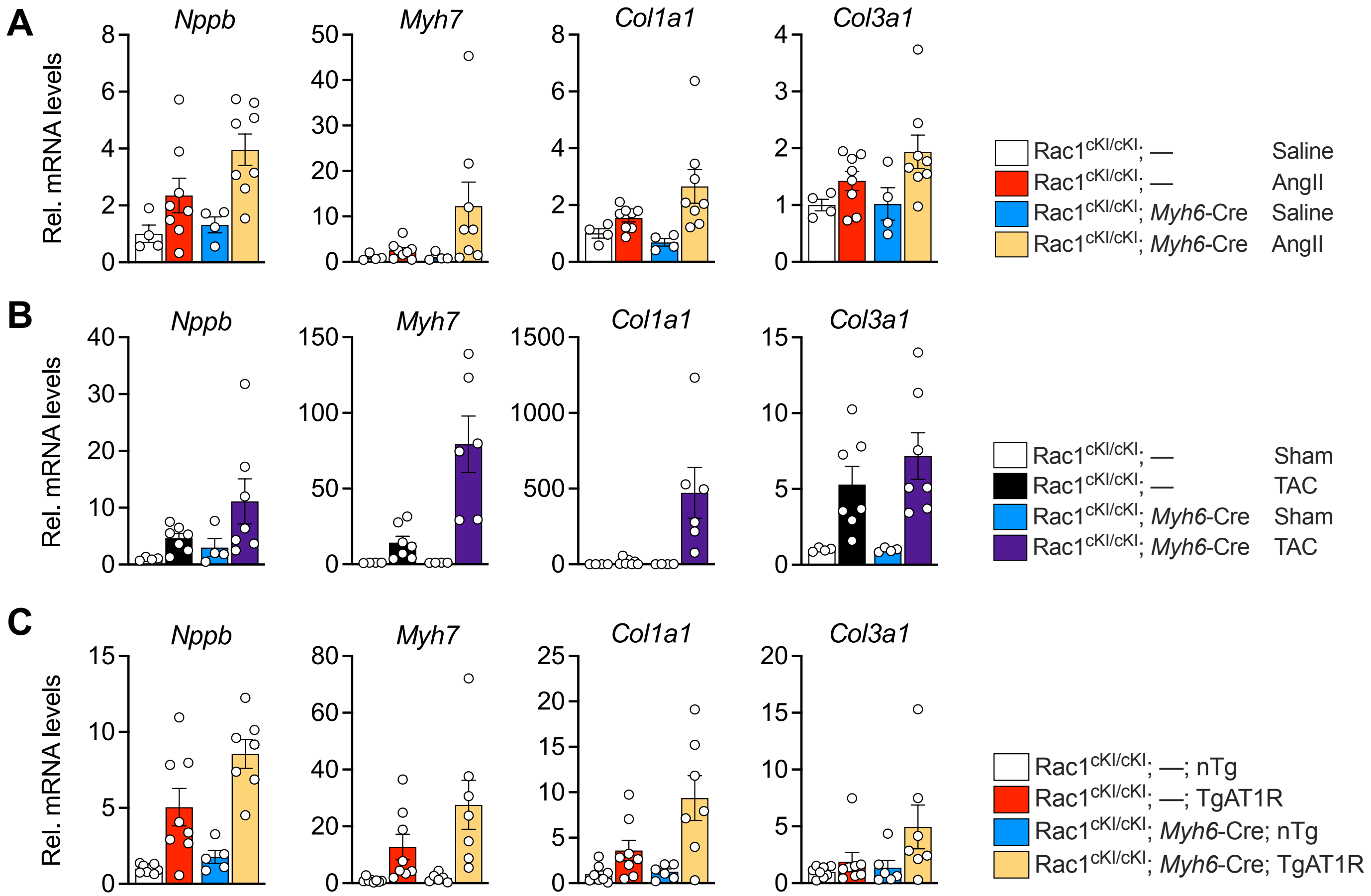

Supplemental Figure 5. Hypertrophic and fibrotic marker gene expression in control and Rac1 conditional knock-in mice in response to models of chronic hypertrophic stress. Relative transcript levels of the hypertrophic marker genes *Nppb* and *Myh7* and fibrotic marker genes *Col1a1* and *Col3a1* were quantified by qPCR in hearts of control or Rac1^{cKI} mice following (**A**) 2 weeks of saline or AngII (3 mg/kg/d) infusion, (**B**) 8 weeks of left ventricular pressure overload induced by transverse aortic constriction (TAC), or (**C**) in 6 months old mice with or without cardiomyocyte-specific transgenic overexpression of the angiotensin-II type I receptor (TgAT1R). n=4-8 per group for **A**, n=4-7 per group for **B**, n=5-8 per group for **C**.

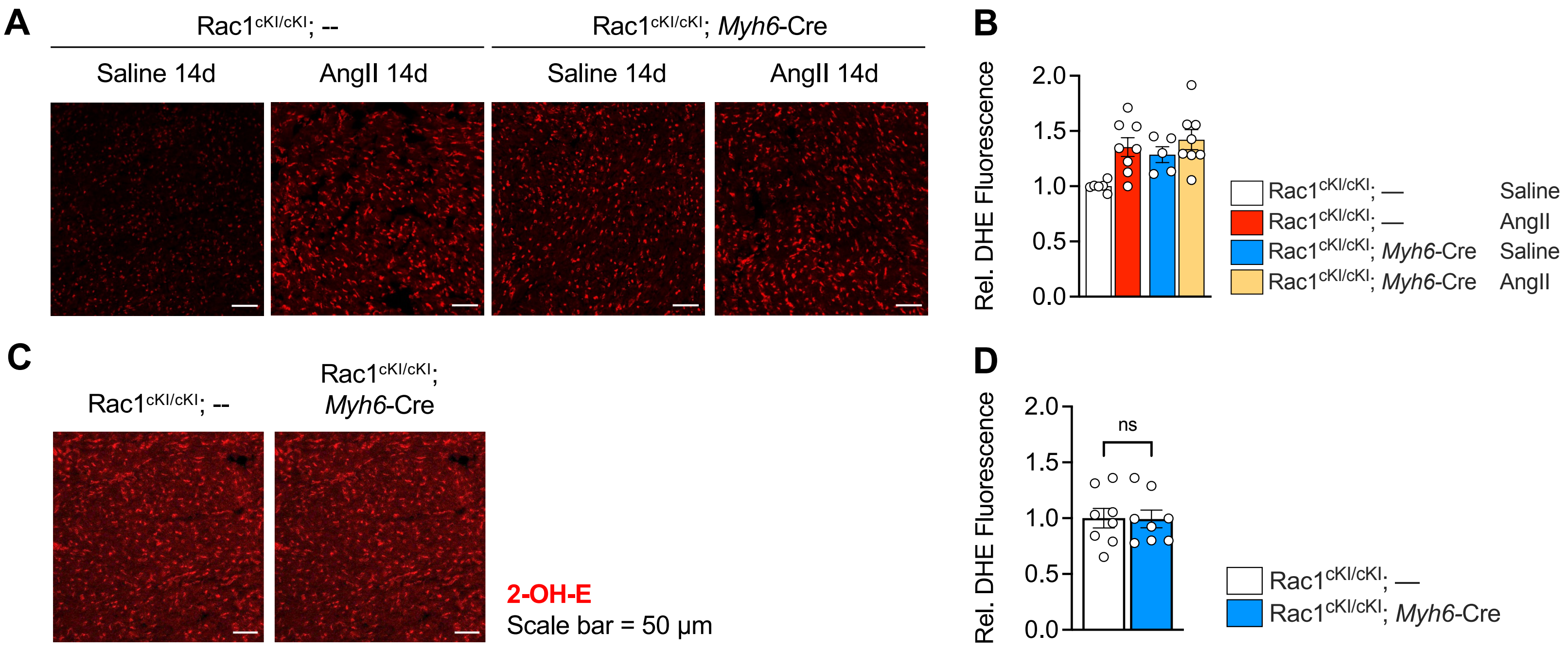

Supplemental Figure 6. Loss of Rac1 cysteine-178 palmitoylation does not affect basal or AngII-stimulated myocardial superoxide levels. (A) Representative DHE-stained, unfixed cardiac sections and **(B)** quantification of relative DHE fluorescence from mice treated with saline or AngII (3 mg/kg/d) for 14 days. NS=not significant by two-way ANOVA. **(C)** Representative DHE-stained, unfixed cardiac sections and **(D)** quantification of relative DHE fluorescence from 2-3-month-old untreated mice of the indicated genotypes. NS=not significant, unpaired t-test, 2-OH-E: 2-hydroxyethidium.

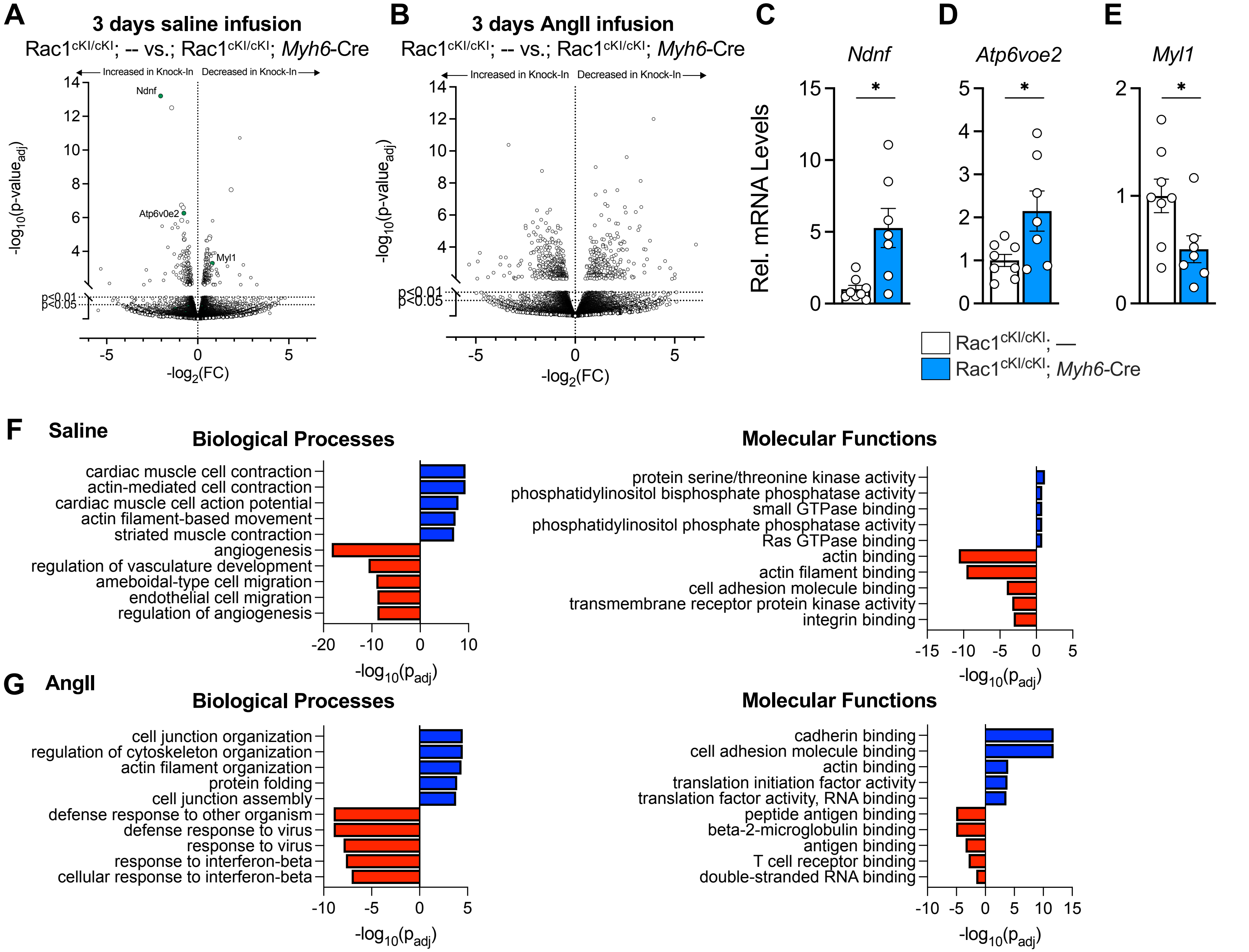

Supplemental Figure 7. RNA sequencing of hearts of saline and AngII-treated control and Rac1 conditional knock-in mice. Volcano plot of differentially expressed genes in hearts of **(A)** saline-treated control vs. saline-treated Rac1^{cKI} mice and **(B)** AngII-treated (3 days, 3 mg/kg/d) control vs. Rac1^{cKI} mice. qPCR validation of the differentially expressed genes **(C)** *Ndnf*, **(D)** *Atp6v0e2*, and **(E)** *Myl1* in saline-treated control vs. Rac1^{cKI} mice. *p<0.05 by unpaired t-test. Histograms of the most differentially upregulated (red) and downregulated (blue) gene ontologies in **(F)** saline-treated Rac1^{cKI} mice compared to saline-treated control mice and **(G)** AngII-treated Rac1^{cKI} mice compared to AngII-treated control mice.

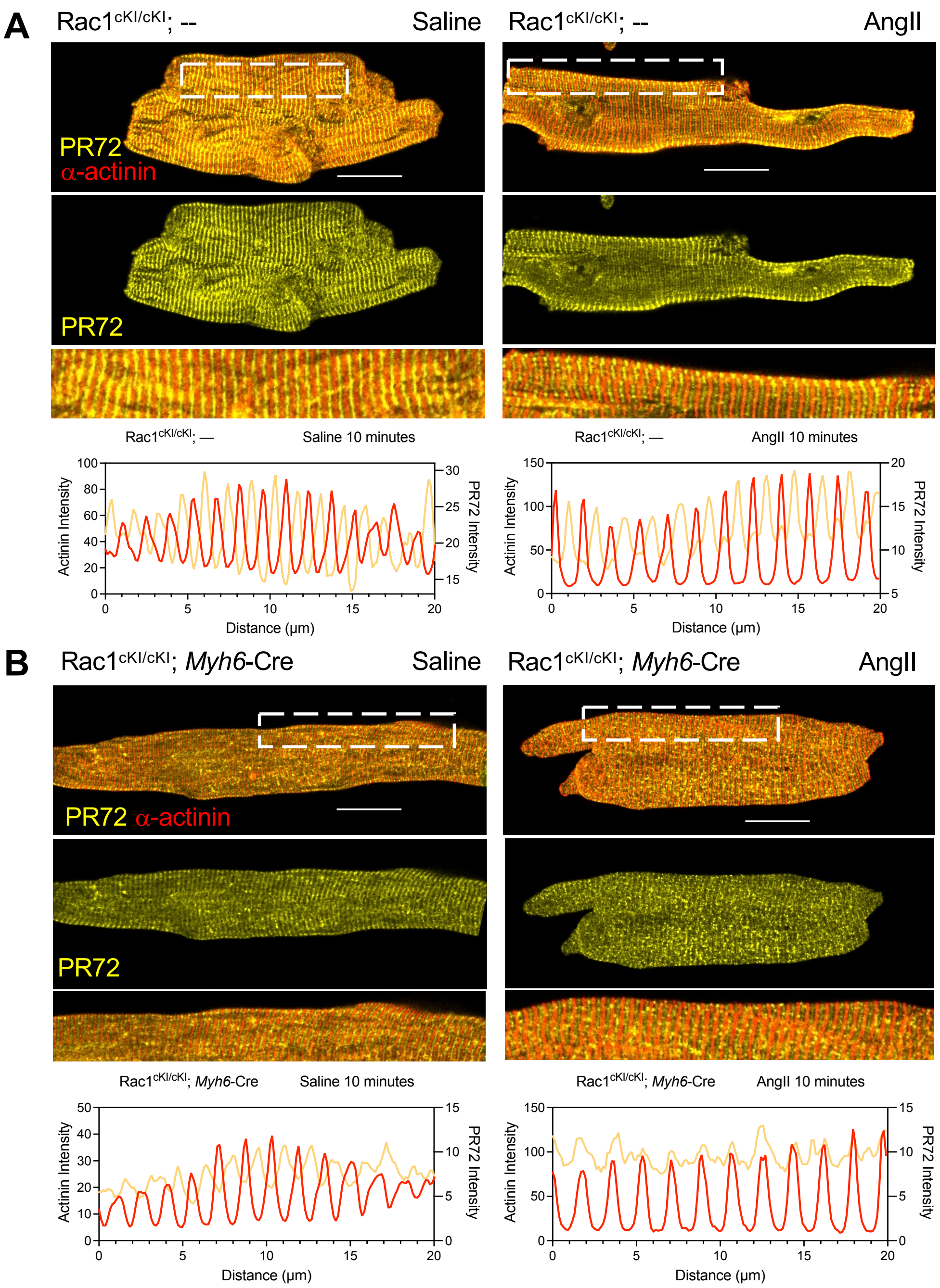

Supplemental Figure 8. PR72/PR130 M-line localization is reduced in Rac1^{cKI} cardiomyocytes. Representative immunocytochemistry of isolated adult cardiomyocytes from (A) control or (B) Rac1^{cKI} mice treated with saline or AngII (10 µM) for 10 minutes. PR72 staining is pseudo-colored yellow and α-actinin staining is pseudo-colored red. Scale bar = 20 µm. Representative plot profiles of PR72 (yellow) and α-actinin (red) intensity are located under each image demonstrating reduced intensity and impaired M-line patterning of PR72 in Rac1^{cKI} myocytes. α-actinin intensity is plotted on the left y-axis and PR72/PR130 intensity is plotted on the right y-axis.

Parameter	*Myh6-Cre*	Rac1$^{cKI/cKI}$; --	Rac1$^{cKI/cKI}$; *Myh6-Cre*
n (M/F)	8 (4/4)	8 (4/4)	8 (4/4)
Heart Rate (bpm)	445.24 ± 72.93	413.50 ± 59.57	418.13 ± 159.28
LVIDs (mm)	2.84 ± 0.60	2.69 ± 0.38	2.77 ± 0.32
LVIDd (mm)	3.81 ± 0.48	3.71 ± 0.42	3.77 ± 0.25
LVESV (µL)	32.54 ± 16.60	27.73 ± 9.72	29.36 ± 8.48
LVEDV (µL)	63.71 ± 19.16	59.75 ± 16.43	61.33 ± 9.26
EF (%)	51.54 ± 10.82	54.32 ± 5.00	52.69 ± 8.26
FS (%)	26.16 ± 6.53	27.61 ± 3.09	26.75 ± 5.11
Stroke Vol. (µL)	29.53 ± 6.17	30.26 ± 8.62	28.53 ± 4.35
CO (mL/min)	13.01 ± 2.66	12.31 ± 2.79	11.76 ± 4.17
Est. LV Mass (mg)	99.03 ± 17.36	100.66 ± 22.35	96.84 ± 11.03
Est. LV/BW (mg/g)	4.11 ± 0.51	4.47 ± 0.69	4.28 ± 0.39
LVAWs (mm)	1.16 ± 0.14	1.20 ± 0.07	1.16 ± 0.12
LVAWd (mm)	0.79 ± 0.08	0.83 ± 0.04	0.79 ± 0.05
LVPWs (mm)	1.03 ± 0.12	1.04 ± 0.12	1.00 ± 0.15
LVPWd (mm)	0.70 ± 0.05	0.72 ± 0.07	0.69 ± 0.05
Body Weight (g)	24.21 ± 4.14	22.35 ± 2.25	22.74 ± 3.05

Supplemental Table 1. Cardiac structural and functional parameters of 4-month-old *Myh6*-Cre, Rac1$^{cKI/cKI}$ or Rac1$^{cKI/cKI}$; *Myh6*-Cre assessed by echocardiography. LVID: left ventricular internal diameter at systole (s) or diastole (d); LVESV: left ventricular end systolic volume; LVEDV: left ventricular end diastolic volume; EF: ejection fraction; FS: fractional shortening; CO: cardiac output, LVAW: left ventricular anterior wall thickness at systole (s) or diastole (d); LVPW: left ventricular posterior wall thickness at systole (s) or diastole (d). Data are presented as mean ± standard deviation. Statistical comparisons by one-way ANOVA with post-hoc Tukey’s multiple comparisons test.

Parameter	*Myh6*-Cre		Rac1$^{cKI/cKI}$; --		Rac1$^{cKI/cKI}$; *Myh6*-Cre		2-way ANOVA p-values		
	Saline	AngII	Saline	AngII	Saline	AngII	Interaction	Drug	Rac1 genotype
n (M/F)	4 (2/2)	8 (4/4)	9 (5/4)	15 (8/7)	9 (4/5)	13 (6/7)	n/a	n/a	n/a
Heart Rate (bpm)	352.36 ± 43.59	420.12 ± 68.00	447.67 ± 93.18	490.07 ± 56.24	432.36 ± 53.69	479.04 ± 78.21	0.8811	0.0110	0.0073
LVIDs (mm)	2.89 ± 0.35	3.05 ± 0.52	2.82 ± 0.25	2.73 ± 0.62	2.70 ± 0.24	3.16 ± 0.67	0.2220	0.2488	0.4899
LVIDd (mm)	3.93 ± 0.28	3.87 ± 0.38	3.79 ± 0.33	3.63 ± 0.45	3.69 ± 0.28	3.89 ± 0.55	0.3883	0.9683	0.4898
LVESV (µL)	32.59 ± 9.00	38.05 ± 15.96	30.63 ± 6.92	29.52 ± 18.79	27.60 ± 6.16	42.52 ± 20.94	0.2510	0.1614	0.5090
LVEDV (µL)	67.36 ± 11.23	65.69 ± 15.11	62.31 ± 13.09	57.48 ± 17.87	58.43 ± 10.58	67.45 ± 22.49	0.3864	0.8627	0.5641
EF (%)	52.29 ± 6.58	43.68 ± 12.25	50.80 ± 4.38	52.16 ± 13.08	52.91 ± 4.40	39.82 ± 13.29	0.0873	0.0323	0.2870
FS (%)	26.53 ± 4.18	21.52 ± 7.04	25.44 ± 2.68	26.63 ± 7.74	26.71 ± 2.70	19.37 ± 7.39	0.0777	0.0421	0.2806
Stroke Vol. (µL)	34.77 ± 3.27	27.65 ± 6.41	26.13 ± 7.12	22.94 ± 5.08	26.83 ± 6.89	21.10 ± 5.50	0.6265	0.0028	0.0048
CO (mL/min)	12.26 ± 1.99	11.37 ± 2.07	11.74 ± 2.24	11.42 ± 2.54	11.38 ± 1.85	10.19 ± 3.56	0.8572	0.2888	0.4718
Est. LV Mass (mg)	92.09 ± 27.81	111.29 ± 17.97	89.75 ± 17.90	123.68 ± 22.62	80.94 ± 13.54	143.09 ± 34.19	0.0406	<0.0001	0.5012
Est. LV/BW (mg/g)	3.64 ± 0.60	5.00 ± 0.88	3.95 ± 0.50	6.16 ± 0.83	3.82 ± 0.31	7.40 ± 1.72	0.0121	<0.0001	0.0055
LVAWs (mm)	1.13 ± 0.12	1.19 ± 0.10	1.03 ± 0.15	1.29 ± 0.18	1.05 ± 0.15	1.23 ± 0.16	0.2397	0.0003	0.8930
LVAWd (mm)	0.73 ± 0.09	0.80 ± 0.09	0.73 ± 0.12	0.93 ± 0.14	0.70 ± 0.13	0.94 ± 0.13	0.1901	<0.0001	0.3599
LVPWs (mm)	0.73 ± 0.14	0.80 ± 0.16	0.92 ± 0.19	1.19 ± 0.20	0.86 ± 0.14	1.10 ± 0.18	0.3274	0.0004	0.0003
LVPWd (mm)	0.61 ± 0.12	0.79 ± 0.15	0.66 ± 0.06	0.91 ± 0.14	0.63 ± 0.08	0.94 ± 0.13	0.3378	<0.0001	0.1287
Body Weight (g)	24.95 ± 3.86	22.50 ± 3.19	22.64 ± 3.36	20.05 ± 2.41	21.18 ± 2.97	19.45 ± 2.71	0.8786	0.0097	0.0120

Supplemental Table 2. Cardiac structural and functional parameters of Rac1$^{cKI/cKI}$ or Rac1$^{cKI/cKI}$; *Myh6*-Cre infused with saline or angiotensin II (3 mg/kg/d) for 2 weeks assessed by echocardiography. LVID: left ventricular internal diameter at systole (s) or diastole (d); LVESV: left ventricular end systolic volume; LVEDV: left ventricular end diastolic volume; EF: ejection fraction; FS: fractional shortening; CO: cardiac output, LVAW: left ventricular anterior wall thickness at systole (s) or diastole (d); LVPW: left ventricular posterior wall thickness at systole (s) or diastole (d). Data are presented as mean ± standard deviation. Statistical comparisons by two-way ANOVA with post-hoc Tukey's multiple comparisons test.

Parameter	*Myh6*-Cre		Rac1$^{cKI/cKI}$; --		Rac1$^{cKI/cKI}$; *Myh6*-Cre		2-way ANOVA p-values		
	Sham	TAC	Sham	TAC	Sham	TAC	Interaction	Surgery	Rac1 genotype
n (M/F)	5 (2/3)	7 (3/4)	6 (3/3)	10 (5/5)	5 (3/2)	9 (4/5)	n/a	n/a	n/a
Heart Rate (bpm)	368.61 ± 52.64	451.51 ± 44.91	379.82 ± 69.11	447.21 ± 45.39	364.98 ± 39.97	397.45 ± 41.91	0.4267	0.0004	0.1906
LVIDs (mm)	2.55 ± 0.12	3.13 ± 0.65	2.75 ± 0.38	3.10 ± 0.38	3.04 ± 0.20	4.05 ± 0.63	0.1996	0.0001	0.0010
LVIDd (mm)	3.69 ± 0.14	4.12 ± 0.48	3.82 ± 0.41	4.08 ± 0.47	4.12 ± 0.20	4.59 ± 0.56	0.7887	0.0090	0.0257
LVESV (µL)	23.62 ± 2.81	41.28 ± 23.29	29.02 ± 9.84	38.90 ± 12.68	36.35 ± 5.78	74.39 ± 27.77	0.1218	0.0005	0.0035
LVEDV (µL)	57.92 ± 5.29	76.63 ± 22.57	63.66 ± 15.86	74.53 ± 21.55	75.19 ± 8.66	98.63 ± 28.39	0.7204	0.0112	0.0392
EF (%)	59.22 ± 3.38	48.72 ± 12.15	55.16 ± 5.00	47.84 ± 6.38	51.61 ± 6.00	25.91 ± 8.64	0.0092	<0.0001	<0.0001
FS (%)	30.80 ± 2.36	24.60 ± 6.93	28.22 ± 3.09	23.86 ± 3.96	26.19 ± 3.68	12.06 ± 4.34	0.0167	<0.0001	<0.0001
Stroke Vol. (µL)	34.30 ± 3.69	35.35 ± 5.21	34.64 ± 6.72	35.63 ± 10.89	38.84 ± 6.94	24.24 ± 6.95	0.0172	0.0939	0.4147
CO (mL/min)	12.52 ± 1.10	16.03 ± 3.12	13.11 ± 3.14	16.11 ± 5.83	14.12 ± 2.58	9.71 ± 3.15	0.0201	0.5710	0.1554
Est. LV Mass (mg)	84.62 ± 14.13	134.86 ± 21.76	95.08 ± 28.09	139.52 ± 38.52	83.94 ± 18.20	156.93 ± 37.69	0.4506	<0.0001	0.6776
Est. LV/BW (mg/g)	3.74 ± 0.37	5.94 ± 0.56	4.28 ± 0.89	5.76 ± 1.16	3.51 ± 0.37	7.41 ± 2.11	0.0440	<0.0001	0.4433
LVAWs (mm)	1.13 ± 0.15	1.40 ± 0.21	1.17 ± 0.24	1.37 ± 0.16	1.05 ± 0.19	1.20 ± 0.17	0.7302	0.0016	0.0840
LVAWd (mm)	0.73 ± 0.16	0.98 ± 0.14	0.78 ± 0.23	0.92 ± 0.13	0.63 ± 0.11	0.90 ± 0.12	0.4364	<0.0001	0.2234
LVPWs (mm)	0.81 ± 0.09	0.81 ± 0.15	0.87 ± 0.08	0.97 ± 0.19	0.70 ± 0.06	0.82 ± 0.20	0.5741	0.1339	0.0296
LVPWd (mm)	0.65 ± 0.11	0.73 ± 0.15	0.65 ± 0.08	0.83 ± 0.19	0.56 ± 0.09	0.76 ± 0.18	0.5573	0.0027	0.3712
Body Weight (g)	22.50 ± 1.82	22.69 ± 2.84	22.05 ± 3.22	24.05 ± 3.62	23.78 ± 3.73	21.44 ± 2.51	0.1900	0.9596	0.9057
Peak Ao Vel (mL/min)	763.07 ± 239.82	3560.46 ± 717.42	933.76 ± 103.21	3858.34 ± 945.18	804.97 ± 219.68	3672.84 ± 918.72	0.9743	<0.0001	0.6875

Supplemental Table 3. Cardiac structural and functional parameters of Rac1$^{cKI/cKI}$ or Rac1$^{cKI/cKI}$; *Myh6*-Cre subjected to sham surgery or transverse aortic constriction (TAC) 8 weeks post-operation assessed by echocardiography. LVID: left ventricular internal diameter at systole (s) or diastole (d); LVESV: left ventricular end systolic volume; LVEDV: left ventricular end diastolic volume; EF: ejection fraction; FS: fractional shortening; CO: cardiac output, LVAW: left ventricular anterior wall thickness at systole (s) or diastole (d); LVPW: left ventricular posterior wall thickness at systole (s) or diastole (d), Ao Vel: aortic velocity. Data are presented as mean ± standard deviation. Statistical comparisons by two-way ANOVA with post-hoc Tukey's multiple comparisons test.

Parameter	$Rac1^{cKI/cKI}$; --		$Rac1^{cKI/cKI}$; *Myh6*-Cre		2-way ANOVA p-values		
	nTg 2 mo	TgAT1R 2 mo	nTg 2 mo	TgAT1R 2 mo	Interaction	AT1R genotype	Rac1 genotype
n (M/F)	10 (6/4)	12 (8/4)	11 (5/6)	8 (5/3)	n/a	n/a	n/a
Heart Rate (bpm)	357.38 ± 48.56	323.24 ± 43.83	336.27 ± 47.14	294.13 ± 30.57	0.7741	0.0091	0.0779
LVIDs (mm)	3.15 ± 0.34	3.56 ± 0.49	2.91 ± 0.32	3.98 ± 0.42	0.0124	<0.0001	0.5065
LVIDd (mm)	4.23 ± 0.36	4.51 ± 0.37	3.93 ± 0.26	4.74 ± 0.35	0.0179	<0.0001	0.7435
LVESV (μL)	40.20 ± 10.95	54.56 ± 16.45	32.99 ± 8.72	70.26 ± 17.23	0.0116	<0.0001	0.3315
LVEDV (μL)	80.75 ± 16.76	93.70 ± 17.93	67.66 ± 10.51	104.96 ± 17.58	0.0202	<0.0001	0.8566
EF (%)	50.65 ± 4.29	42.63 ± 10.62	51.76 ± 7.19	33.75 ± 7.02	0.0507	<0.0001	0.1251
FS (%)	25.56 ± 2.57	21.18 ± 6.49	26.23 ± 4.51	16.12 ± 3.64	0.0623	<0.0001	0.1484
Stroke Volume (μL)	40.55 ± 6.82	39.13 ± 8.67	34.67 ± 4.70	34.70 ± 5.72	0.7372	0.7488	0.0210
CO (mL/min)	14.46 ± 2.91	12.75 ± 3.76	11.75 ± 2.23	10.12 ± 1.51	0.9203	0.0785	0.0043
Est. LV Mass (mg)	103.08 ± 19.12	109.04 ± 22.05	92.66 ± 18.93	100.92 ± 16.57	0.8534	0.2576	0.1422
Est. LV/BW (mg/g)	3.69 ± 0.43	3.94 ± 0.60	3.42 ± 0.45	3.67 ± 0.52	0.9974	0.1321	0.0986
LVAWs (mm)	1.12 ± 0.15	1.08 ± 0.11	1.14 ± 0.17	0.95 ± 0.18	0.1292	0.0155	0.2392
LVAWd (mm)	0.76 ± 0.15	0.71 ± 0.09	0.74 ± 0.13	0.63 ± 0.13	0.4596	0.0453	0.2616
LVPWs (mm)	0.75 ± 0.07	0.74 ± 0.20	0.74 ± 0.11	0.60 ± 0.13	0.1487	0.1121	0.0964
LVPWd (mm)	0.58 ± 0.06	0.58 ± 0.12	0.61 ± 0.08	0.50 ± 0.10	0.0780	0.0510	0.4184
Body Weight (g)	22.42 ± 3.57	22.33 ± 3.85	21.68 ± 3.55	22.14 ± 3.02	0.8076	0.8733	0.6825

Supplemental Table 4. Cardiac structural and functional parameters of 2-month-old $Rac1^{cKI/cKI}$ or $Rac1^{cKI/cKI}$; *Myh6*-Cre mice with or without transgenic AT1R expression assessed by echocardiography. LVID: left ventricular internal diameter at systole (s) or diastole (d); LVESV: left ventricular end systolic volume; LVEDV: left ventricular end diastolic volume; EF: ejection fraction; FS: fractional shortening; CO: cardiac output, LVAW: left ventricular anterior wall thickness at systole (s) or diastole (d); LVPW: left ventricular posterior wall thickness at systole (s) or diastole (d). Data are presented as mean ± standard deviation. Statistical comparisons by two-way ANOVA with post-hoc Tukey's multiple comparisons test.

Parameter	Rac1$^{cKI/cKI}$; --		Rac1$^{cKI/cKI}$; *Myh6*-Cre		2-way ANOVA p-values		
	nTg 4 mo	TgAT1R 4 mo	nTg 4 mo	TgAT1R 4 mo	Interaction	AT1R genotype	Rac1 genotype
n (M/F)	12 (6/6)	11 (7/4)	9 (2/7)	11 (6/6)	n/a	n/a	n/a
Heart Rate (bpm)	393.94 ± 55.44	348.56 ± 31.70	380.45 ± 62.36	309.73 ± 49.06	0.4174	0.0006	0.0985
LVIDs (mm)	2.71 ± 0.46	3.61 ± 0.53	2.51 ± 0.38	4.58 ± 0.81	0.0020	<0.0001	0.0342
LVIDd (mm)	3.90 ± 0.30	4.55 ± 0.50	3.71 ± 0.29	5.31 ± 0.65	0.0020	<0.0001	0.0504
LVESV (µL)	28.41 ± 11.15	56.54 ± 19.00	23.35 ± 9.01	100.24 ± 40.21	0.0017	<0.0001	0.0111
LVEDV (µL)	66.36 ± 12.55	96.56 ± 23.61	59.15 ± 10.91	138.68 ± 37.79	0.0018	<0.0001	0.0230
EF (%)	58.48 ± 10.62	42.43 ± 8.63	61.11 ± 10.47	29.90 ± 10.26	0.0181	<0.0001	0.1148
FS (%)	30.96 ± 7.53	20.93 ± 4.94	32.57 ± 7.13	14.29 ± 5.25	0.0391	<0.0001	0.2012
Stroke Volume (µL)	37.96 ± 5.16	40.02 ± 8.98	35.80 ± 8.09	38.43 ± 8.81	0.9066	0.3357	0.4419
CO (mL/min)	15.05 ± 3.35	14.05 ± 3.72	13.90 ± 4.96	11.95 ± 3.42	0.6909	0.2190	0.1750
Est. LV Mass (mg)	104.03 ± 34.08	122.64 ± 27.07	86.89 ± 10.98	134.14 ± 29.38	0.0986	0.0004	0.7407
Est. LV/BW (mg/g)	4.09 ± 0.88	4.79 ± 0.62	3.78 ± 0.25	5.48 ± 1.05	0.0441	<0.0001	0.4150
LVAWs (mm)	1.22 ± 0.25	1.08 ± 0.14	1.26 ± 0.18	0.96 ± 0.15	0.2095	0.0005	0.4872
LVAWd (mm)	0.81 ± 0.16	0.73 ± 0.10	0.78 ± 0.09	0.66 ± 0.10	0.5853	0.0128	0.1526
LVPWs (mm)	0.78 ± 0.20	0.74 ± 0.10	0.77 ± 0.18	0.63 ± 0.15	0.3431	0.0631	0.2535
LVPWd (mm)	0.68 ± 0.15	0.65 ± 0.06	0.62 ± 0.03	0.54 ± 0.10	0.3731	0.0969	0.0120
Body Weight (g)	25.30 ± 4.35	25.62 ± 4.38	23.13 ± 4.09	24.57 ± 3.85	0.6674	0.4998	0.2193

Supplemental Table 5. Cardiac structural and functional parameters of 4-month-old Rac1$^{cKI/cKI}$ or Rac1$^{cKI/cKI}$; *Myh6*-Cre mice with or without transgenic AT1R expression assessed by echocardiography. LVID: left ventricular internal diameter at systole (s) or diastole (d); LVESV: left ventricular end systolic volume; LVEDV: left ventricular end diastolic volume; EF: ejection fraction; FS: fractional shortening; CO: cardiac output, LVAW: left ventricular anterior wall thickness at systole (s) or diastole (d); LVPW: left ventricular posterior wall thickness at systole (s) or diastole (d). Data are presented as mean ± standard deviation. Statistical comparisons by two-way ANOVA with post-hoc Tukey's multiple comparisons test.

Parameter	Rac1$^{cKI/cKI}$; --		Rac1$^{cKI/cKI}$; *Myh6*-Cre		2-way ANOVA p-values		
	nTg 6 mo	TgAT1R 6 mo	nTg 6 mo	TgAT1R 6 mo	Interaction	AT1R genotype	Rac1 genotype
n (M/F)	15 (7/8)	11 (5/6)	10 (3/7)	12 (6/6)	n/a	n/a	n/a
Heart Rate (bpm)	391.29 ± 37.09	332.38 ± 23.07	409.15 ± 59.70	316.44 ± 76.24	0.2748	<0.0001	0.9503
LVIDs (mm)	2.92 ± 0.48	3.68 ± 0.40	2.95 ± 0.46	5.89 ± 1.37	<0.0001	<0.0001	<0.0001
LVIDd (mm)	4.00 ± 0.38	4.57 ± 0.41	3.94 ± 0.35	6.37 ± 1.27	<0.0001	<0.0001	0.0001
LVESV (µL)	34.27 ± 13.62	58.34 ± 16.05	34.89 ± 13.43	184.09 ± 84.54	<0.0001	<0.0001	<0.0001
LVEDV (µL)	70.96 ± 15.89	96.70 ± 22.01	68.23 ± 14.77	216.62 ± 88.52	<0.0001	<0.0001	<0.0001
EF (%)	53.15 ± 9.83	40.07 ± 5.60	50.04 ± 11.09	17.92 ± 9.85	0.0011	<0.0001	<0.0001
FS (%)	27.30 ± 6.20	19.51 ± 3.19	25.39 ± 6.83	8.36 ± 4.77	0.0058	<0.0001	0.0002
Stroke Volume (µL)	36.69 ± 6.27	38.36 ± 7.86	33.35 ± 6.50	32.53 ± 9.45	0.5758	0.8482	0.0442
CO (mL/min)	14.41 ± 3.16	12.75 ± 2.76	13.82 ± 4.11	9.90 ± 2.94	0.2407	0.0051	0.0765
Est. LV Mass (mg)	97.65 ± 19.88	119.31 ± 19.19	94.82 ± 15.79	171.73 ± 53.68	0.0042	<0.0001	0.0095
Est. LV/BW (mg/g)	3.77 ± 0.49	4.88 ± 0.44	3.97 ± 0.58	7.73 ± 2.67	0.0023	<0.0001	0.0006
LVAWs (mm)	1.20 ± 0.19	1.12 ± 0.07	1.10 ± 0.14	0.83 ± 0.14	0.0419	0.0002	<0.0001
LVAWd (mm)	0.74 ± 0.15	0.75 ± 0.08	0.71 ± 0.12	0.57 ± 0.08	0.0203	0.0516	0.0024
LVPWs (mm)	0.73 ± 0.13	0.73 ± 0.08	0.76 ± 0.10	0.64 ± 0.11	0.0911	0.0625	0.3594
LVPWd (mm)	0.64 ± 0.11	0.61 ± 0.07	0.67 ± 0.09	0.54 ± 0.09	0.1172	0.0041	0.5342
Body Weight (g)	25.95 ± 4.25	24.65 ± 4.72	24.12 ± 4.21	22.43 ± 3.25	0.8730	0.2224	0.0998

Supplemental Table 6. Cardiac structural and functional parameters of 6-month-old Rac1$^{cKI/cKI}$ or Rac1$^{cKI/cKI}$; *Myh6*-Cre mice with or without transgenic AT1R expression assessed by echocardiography. LVID: left ventricular internal diameter at systole (s) or diastole (d); LVESV: left ventricular end systolic volume; LVEDV: left ventricular end diastolic volume; EF: ejection fraction; FS: fractional shortening; CO: cardiac output, LVAW: left ventricular anterior wall thickness at systole (s) or diastole (d); LVPW: left ventricular posterior wall thickness at systole (s) or diastole (d). Data are presented as mean ± standard deviation. Statistical comparisons by two-way ANOVA with post-hoc Tukey’s multiple comparisons test.

Gene	Species	Forward	Reverse
Acta1	Mouse	ACGCCAGCCTCTGAAACTAGA	GCCGTTGTCACACACAAGAG
Atp6v0e2	Mouse	TCTGCTGTTACCTCTTCTGGC	GCACGTACCAGATGGTCTCA
Col1a1	Mouse	AGGTATGCTTGATCTGTAT	CAGTCCAGTTCTTCATTG
Col3a1	Mouse	CTGTAACATGGAAACTGGGGAAA	CCATAGCTGAACTGAAAACCACC
Gapdh	Mouse	CTGCTTCAGGGAGACACACC	TGTGGTCATGAGCCCTTCC
Myh7	Mouse	CAGCCATGCCAACCGTATG	TTCCACGATGGCGATGTTC
Myl1	Mouse	AAGATCGAGTTCTCTAAGGAGCA	TCATGGGCAGAAACTGTTCAAA
Ndnf	Mouse	CGATTCATCTGTGATTCCA	GTGTTATCTTCCTCAACCAT
Nppa	Mouse	TTCTTCCTCGTCTTGGCCTTT	GACCTCATCTTCTACCGGCATCT
Nppb	Mouse	CACCGCTGGGAGGTCACT	GTGAGGCCTTGGTCCTTCAAGGTCACT
Postn	Mouse	CTGCTTCAGGGAGACACACC	TCTGGCCTCTGGGTTTTCAC
Ppp2r3a (PR130-specific)	Mouse	CATTATAGCAGTGTAGTGATAGAC	GTGAAGGTATGGCAGGTT
Ppp2r3a (PR72-specific)	Mouse	TGAGCAGACGTTGCGAGTCA	TGGGACCACAATCCATGCTT

Supplemental Table 7. Sequences of primers used for RT-qPCR.
