# Supplementary material for: Rac1 palmitoylation is required for cardiac stress adaptation and regulation of protein kinase A signaling
Source: JCI Insight. 2025 Sep 9;10(20):e193733. doi: 10.1172/jci.insight.193733 (PMC12581661; doi:10.1172/jci.insight.193733)

Figure 1

Fig 1B

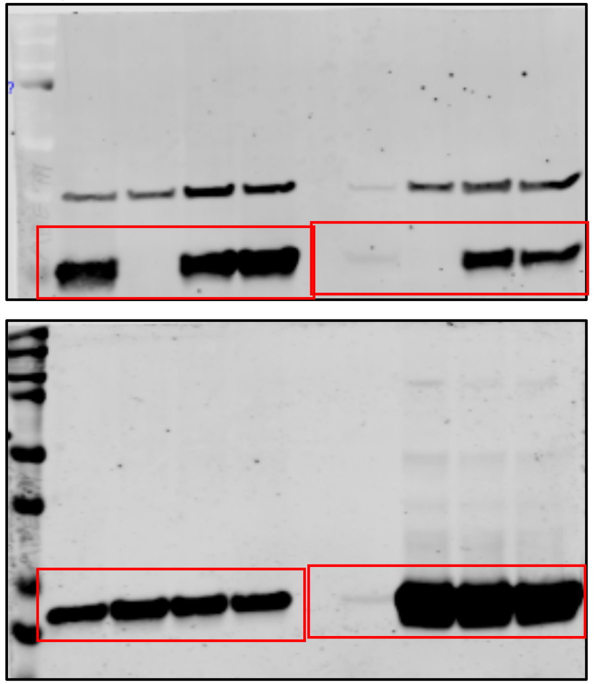

Fig 1D

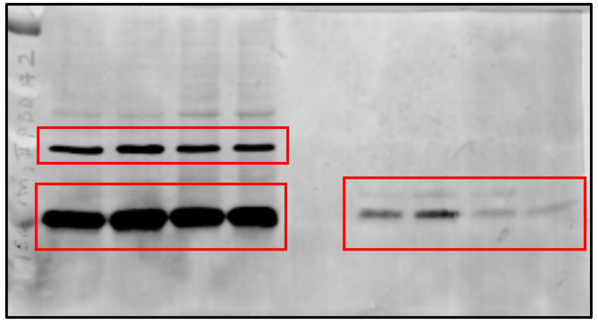

Fig 1F

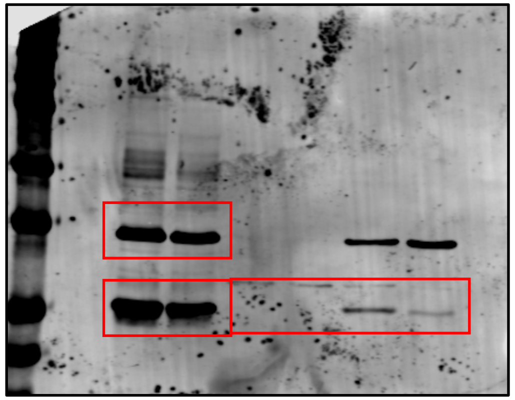

Fig 1G

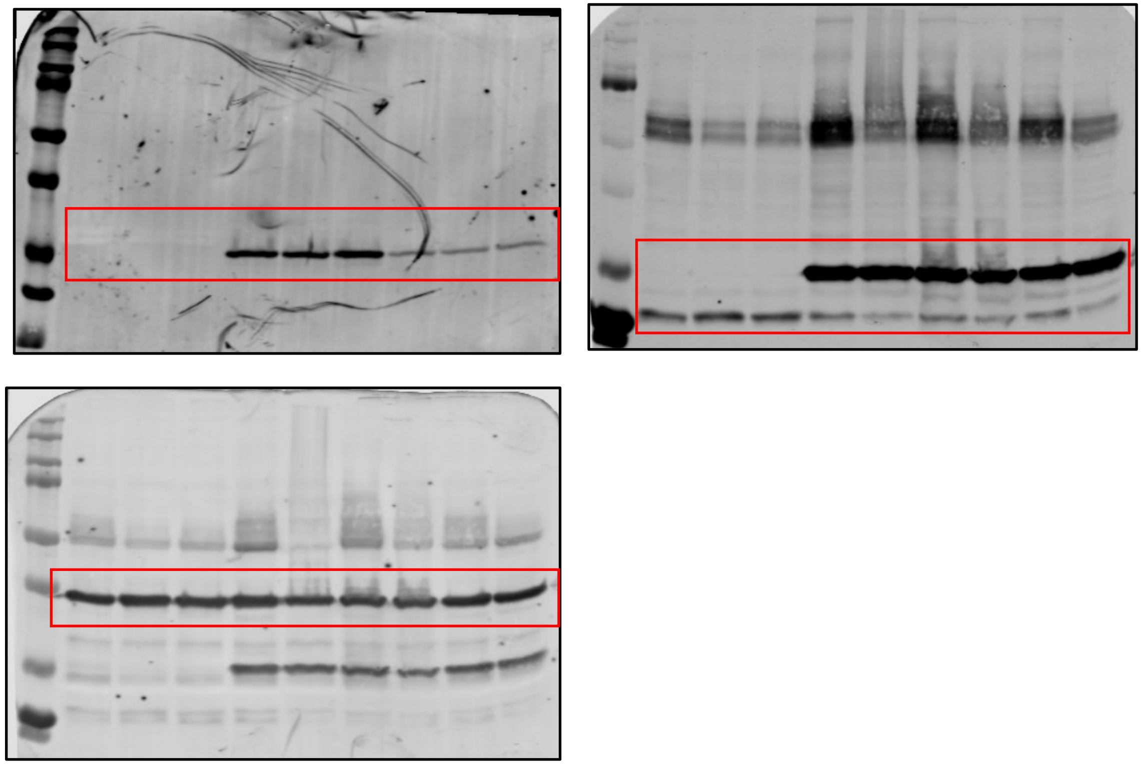

Figure 1 (cont.)

Fig 1I

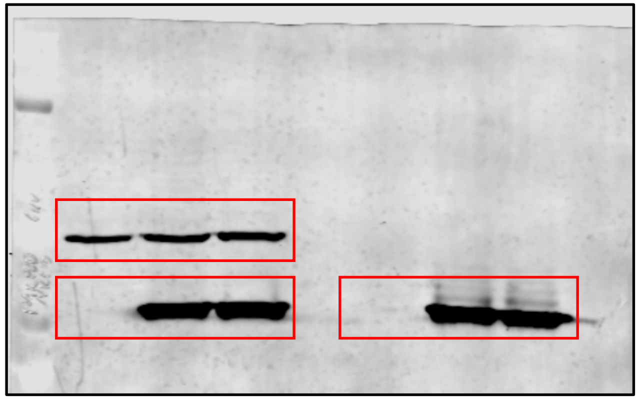

Figure 2

Fig 2B

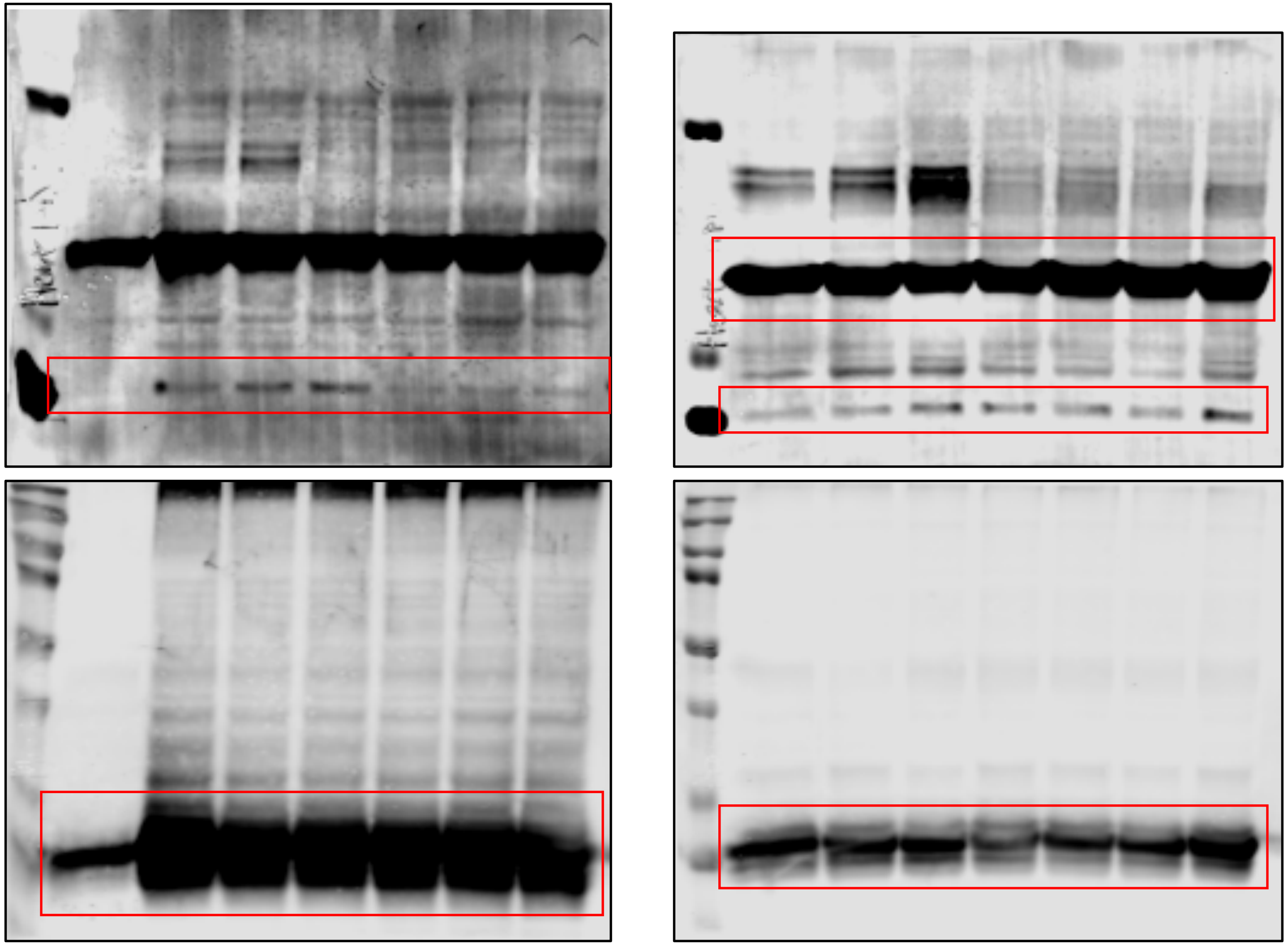

Figure 6

Fig 6A

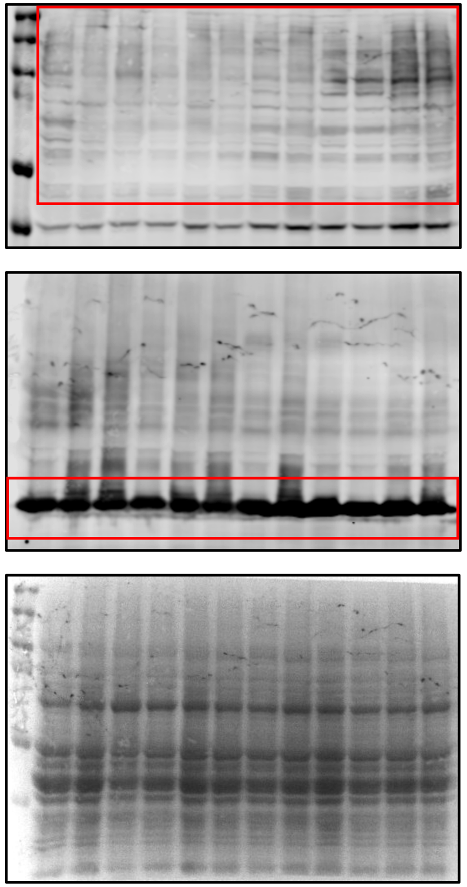

Fig 6C

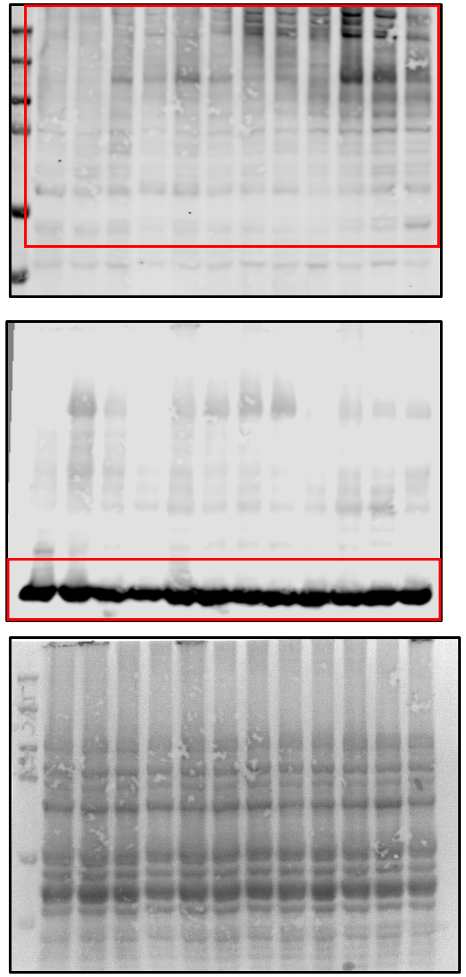

Fig 6E

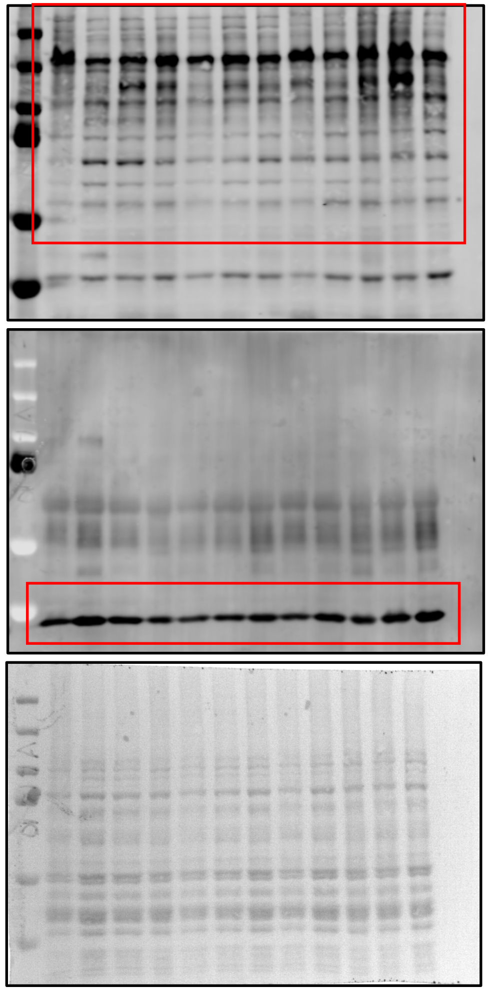

Figure 7

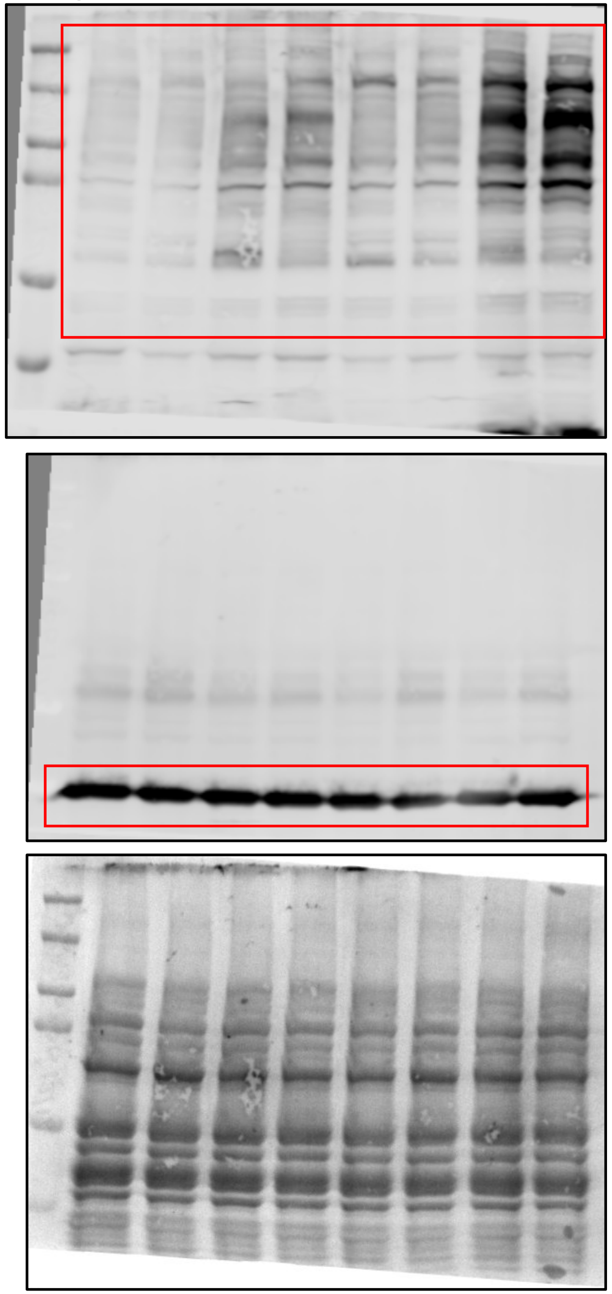

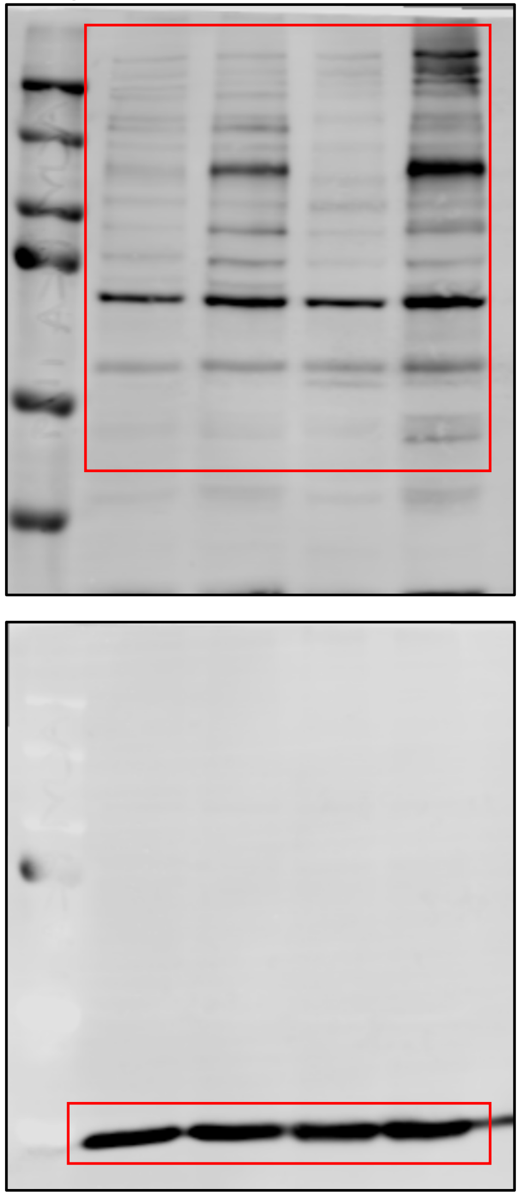

Figure 8

Fig 8E

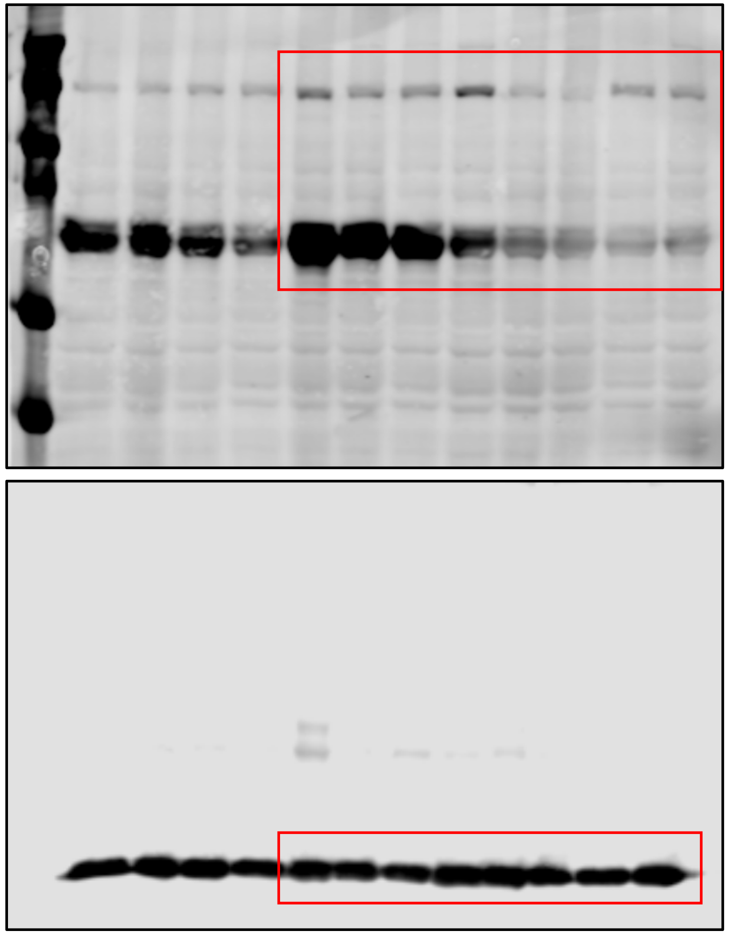

Supplemental Figure 2

Fig S2A

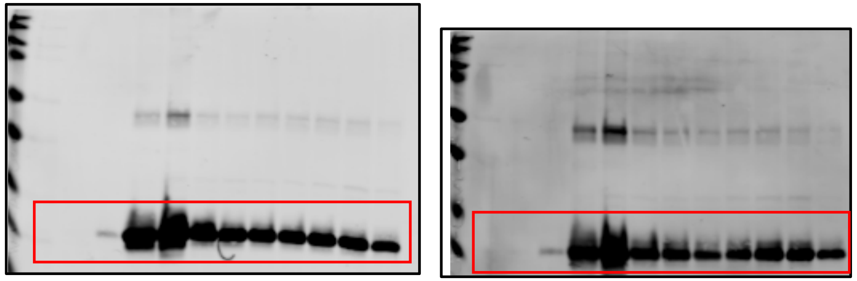

Fig S2B

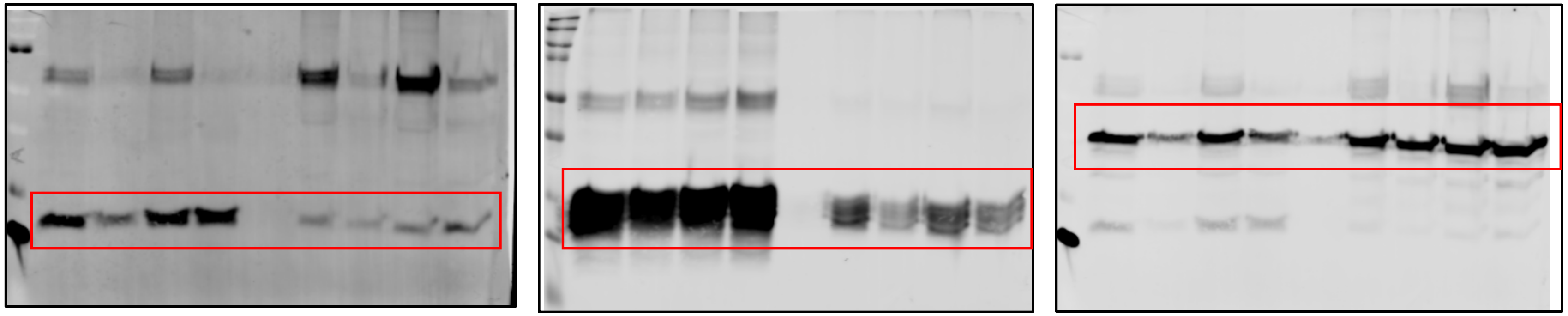

Supplemental Figure 2

Fig S2E

Cytosolic Fractions

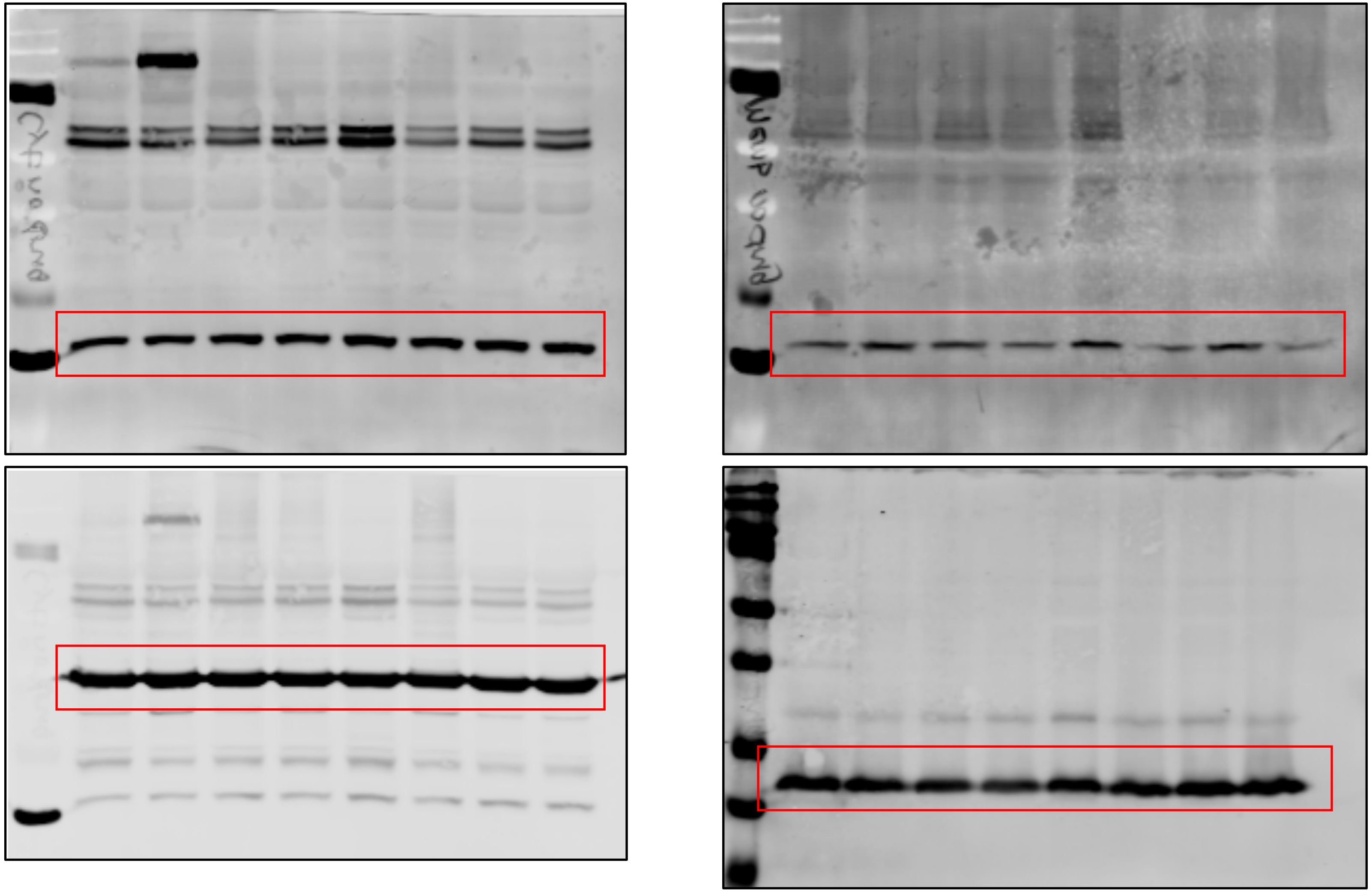

Supplement: Unedited blot and gel images [file jciinsight-10-193733-s056.pdf]
